# Effects of color cues on eye-hand coordination training with a mirror drawing task in virtual environment

**DOI:** 10.3389/fpsyg.2023.1307590

**Published:** 2024-01-15

**Authors:** Zainab Alrubaye, Moaaz Hudhud Mughrabi, Banu Manav, Anil Ufuk Batmaz

**Affiliations:** ^1^Architecture Department, Art and Design Faculty, Kadir Has University, Istanbul, Türkiye; ^2^Mechatronics Engineering Department, Faculty of Engineering and Natural Sciences, Kadir Has University, Istanbul, Türkiye; ^3^Interior Architecture and Environmental Design Department, Art and Design Faculty, Kadir Has University, Istanbul, Türkiye; ^4^Computer Science and Software Engineering Department, Gina Cody School of Engineering and Computer Science, Concordia University, Montreal, QC, Canada

**Keywords:** virtual reality, mirror drawing test, eye-hand coordination, training, motor performance, color, color theory

## Abstract

Mirror drawing is a motor learning task that is used to evaluate and improve eye-hand coordination of users and can be implemented in immersive Virtual Reality (VR) Head-Mounted Displays (HMDs) for training purposes. In this paper, we investigated the effect of color cues on user motor performance in a mirror-drawing task between Virtual Environment (VE) and Real World (RW), with three different colors. We conducted a 5-day user study with twelve participants. The results showed that the participants made fewer errors in RW compared to VR, except for pre-training, which indicated that hardware and software limitations have detrimental effects on the motor learning of the participants across different realities. Furthermore, participants made fewer errors with the colors close to green, which is usually associated with serenity, contentment, and relaxation. According to our findings, VR headsets can be used to evaluate participants' eye-hand coordination in mirror drawing tasks to evaluate the motor-learning of participants. VE and RW training applications could benefit from our findings in order to enhance their effectiveness.

## 1 Introduction

Immersive Virtual Reality (VR) Head Mounted Displays (HMDs) provide a digitally generated environment that immerses users in a simulated environment. The term (virtual) in VR refers to something made virtually out of software rather than being real and physical; a virtual object is more flexible than its physical twin (Soliman et al., 2021). Scenarios created in the virtual world follow a similar creative process to those created in the real world. An alternate world might be a representation of an actual space that exists elsewhere or might be purely fictional (Sherman and Craig, 2018). It could be a world that is vastly different from our own or one with subtle variations. It can also be a virtual world in which certain events and outcomes differ from the real world while representing it.

One of the main advantages of VR lies in its ability to provide individuals with a (relatively) secure and cost-effective platform to perform tasks (Hamad and Jia, 2022). Within an immersive virtual world, users, such as technicians, athletes, or surgeons, can undergo training that generates a high level of presence, where they feel as if they are physically present in that virtual environment, experiencing a strong sense of realism and engagement. Due to its ability to provide immersive and interactive learning experiences, VR is increasingly becoming an important component of contemporary learning methodologies (Hansen et al., 2008). Empirical evidence also suggests that a virtual environment exhibits the ability to facilitate learning and comprehension due to its potential to establish a robust integration between symbolic representations and experiential information (Bowman et al., 1999). One of the particularly important domains of training is motor learning, also known as procedural memory, which encompasses the acquisition and refinement of skills that involve physical movement. Learners can develop new skills and gain a better understanding of the subject by integrating virtual reality technology into their learning process. One of the testbeds used to evaluate motor learning of participants is the mirror drawing test, which is used to investigate the acquisition of learned motor skills under varying contextual factors (Bowman et al., 1999; Alizadehsalehi et al., 2019; Hamad and Jia, 2022).

In addition to in real life, in VR, color plays a vital role in creating a realistic and immersive environment (Carruth, 2017; Natephra et al., 2017). Color can be used to affect the mood and emotions of learners in a VR environment, enhancing the learning experience (Um et al., 2012). Therefore, when designing VR experiences for education, color should be carefully considered to ensure that it supports the learning objectives and creates an engaging and effective learning environment. Moreover, color can have a significant impact on our sensory systems in a VR environment, and its use can help us create different immersive experiences. Color can be used to manipulate our visual attention and guide our movement through the environment. By choosing the right colors, it is possible to create an immersive experience that encourages learners to explore and interact with the environment.

In this paper, we hypothesize that with warm color cues, such as red, participants will be faster and make fewer errors in virtual environments for eye-hand coordination training tasks, due to the immersive and interactive nature of the technology. We also hypothesize that there is a difference in user performance and learning curve between the virtual environment (VE) and real world (RW), with VE environment showing a steeper learning curve and potentially higher performance level, because of the hardware limitations of the VR headset, such as stereo deficiencies (Batmaz et al., 2022). This hypothesis assumes that the simulated environment provided by VR technology may allow for more controlled and consistent training conditions, which could lead to faster skill possession and higher levels of execution. To test our hypotheses, we used a mirror-drawing test since it requires precise coordination between visual input, e.g., the different color used in this study, and motor output, the drawings.

This study aims to investigate how color cues influence training in two separate settings: the real world and the virtual world. These environments are carefully replicated to mirror similar physical conditions, including objects, lighting, and the task at hand. Specifically, the study seeks to investigate how the use of color cues affects training outcomes in VR compared to traditional real-life training in a mirror drawing task.

## 2 Previous work

### 2.1 Mirror drawing test

Mirror drawing is one of the tests to investigate the acquisition of learned motor skills (Merbah and Meulemans, 2011), the range of behavior (Ellis et al., 1957; Salowitz et al., 2013), and cognitive systems such as eye-hand coordination (Kline, 1937; Bhushan et al., 2000). In mirror drawing tasks, participants replicate or trace an existing pattern or image by looking at the mirror's reflection.

To complete the task, participants are required to coordinate their visual perception and manual dexterity accurately (Carmichael, 1927). The consistent practice of mirror drawing exercises improves the eye-hand coordination of the participant, while enhancing the synchronization and alignment between visual information processing and motor movements (Marks, 1996). The mirror serves as a feedback tool and enables participants to directly observe the relationship between their hand dexterity and the corresponding visual results in the mirror.

Through repetitive mirror drawing tasks, participants can develop and refine their fine motor skills, spatial awareness, and eye-hand coordination. The process involves continuous adjustments and corrections based on the visual feedback provided by the reflection of the mirror. Over time, these iterative refinements contribute to incremental advancements in execution time and accuracy.

As eye-hand coordination highly depends on visual stimuli to guide the hand as it moves, previous research also suggests that visual factors, such as color, have an effect on eye-hand coordination (Acirli et al., 2022).

### 2.2 Effect of colors on motor skills

One of the focuses of color theory is how color affects emotions and perceptions of individuals. Colors can evoke different emotional and cognitive responses, which can influence motor performance and coordination (Tenenbaum et al., 2009; Balakrishnan et al., 2014). A warm color, such as red or yellow, can evoke energy and excitement, whereas a cool color, such as blue or green, can evoke a feeling of calmness and peace. Red has been associated with excitement, orange with distress and upset, purple with dignity and stateliness, yellow with joy, and blue with security and comfort (Kaya and Epps, 2004). Therefore, colors and emotions are closely intertwined, and color has been widely recognized as a powerful factor in influencing moods, feelings, and emotions (Cipresso et al., 2018; Hong et al., 2022).

As color can evoke emotions ranging from joy and excitement to sadness and anger, emotions, in turn, can also affect fine motor skills or manual dexterity, e.g., when a person is anxious or nervous, their hands can tremble, leading to changes in the quality of handwriting. On the contrary, a calm and focused emotional state can lead to more precise fine motor control (Ayzeren et al., 2019). For example, in activities that require fine motor skills, such as playing a musical instrument or performing surgery, the emotional state of the individual can affect their precision, speed, and accuracy (Gray, 2001). Positive emotions, such as happiness or excitement, can improve fine motor skills, while negative emotions, such as sadness or frustration, can hinder them (Cheung et al., 2022). Pleasant and unpleasant stimuli also have an effect on the coordination between visual information and motor control, where unpleasant emotions increase the possibility of error and rapid movement (Coombes et al., 2005). Furthermore, emotional states can affect the rate of fine motor skill acquisition and learning (Taravati et al., 2022), e.g., a person who is relaxed and positive may learn fine motor skills more quickly and effectively than someone who is anxious or stressed. Emotions can also influence overall task performance.

The color of our surroundings goes beyond influencing emotions; it also plays a crucial role in shaping perception, affecting the performance and fatigue levels during visual tasks. Cool colors, leaning toward blue tones, have been identified as a contributing factor in reducing visual strain and enhancing performance accuracy in visual tracking tasks (Jiang et al., 2023). Additionally, the vibrancy of colors does not only affect emotions but also plays a role in determining human performance and physiology (Al-Ayash et al., 2016). Thus, it is important to understand how color influences performance when training for hand-eye coordination skills.

The interplay between color and motor skills is complex and includes psychological and environmental factors. Environmental colors can influence arousal and attention, with bright hues fostering alertness and quicker motor responses, while softer tones may promote focus and control in precision tasks (Weijs et al., 2023). In addition, color-coded systems, widely used in offices and industrial settings, enhance organization and coordination, helping to provide efficient motor responses (Rondinelli and Vastag, 2000). Universally recognized safety colors, such as red and green, trigger rapid responses in contexts like traffic lights or emergency vehicles (Julius and Adi-Japha, 2016). Moreover, individual responses to colors, influenced by personal preferences and cultural factors, can affect motivation and mood, indirectly influencing engagement and motor performance (Palmer et al., 2013). Recognizing these relationships allows the creation of environments that optimize motor skill development and performance.

### 2.3 Real world vs. virtual worlds

One of the key features of VR HMDs is their ability to simulate complex real-world tasks without requiring specialized equipment. This innovative capability is particularly valuable in fields like medical training, where surgical simulations provide a relatively risk-free environment for practitioners to practice and refine intricate procedures while mitigating the use of expensive training materials (Ellis, 1994; Farra et al., 2019). This not only improves professional skills and competency, but also minimizes potential risks associated with real-life surgeries. This approach also facilitates comprehensive skill development and improves proficiency in other fields, such as aviation, manufacturing, and emergency response training.

Previous research has demonstrated that training in virtual environments can effectively transfer to real-world skills across various tasks (Rose et al., 2000), particularly due to its flexibility and realism (Michalski et al., 2019, Levac et al., 2019). In addition, virtual training allows users to customize their practice to focus on the skills they need, such as time or error rate.

However, the motor performance of users is affected in the virtual environment compared to the real world, influencing performance and accuracy during fine motor tasks (Arnold et al., 2002). This discrepancy can be explained by the presence of stereo deficiencies in the virtual environment (Barrera Machuca and Stuerzlinger, 2019), such as the vergence-accommodation conflict (Hoffman et al., 2008; Barrera Machuca and Stuerzlinger, 2019; Batmaz et al., 2022) or diplopia (Bruder et al., 2013). For example, Barrera Machuca and Stuerzlinger (2019) compared user selection performance in both real-world and virtual environments. Their study revealed that state-of-the-art stereodisplays significantly reduce user performance compared to real-world setups. In this paper, we are also investigating how the different hardware and software limitations of VR HMDs vary the effect of colors on human motor learning.

## 3 Materials and methods

### 3.1 Participants

We post flyers, send emails, and send messages to reach out to colleagues, professors, or industry professionals who may have access to potential participants. We also get in touch with universities and academic institutions to recruit participants from different student populations. We also promoted our work on social media platforms to invite participants. As a result, 12 participants (four male and eight female) between 19 and 31 years of age (average was 22) participated in this study. We performed the Ishihara Color Blindness Test (Clark, 1924) before starting the experiment, and none of the participants was color-blind. Nine out of 12 had normal vision, while three participants had corrected to normal vision. All participants were right-handed according to the Edinburg Inventory (Oldfield, 1971), and the dominant eye of nine of 12 participants was right. No participant had an injury or disability that could affect their participation in the study.

### 3.2 Apparatus

#### 3.2.1 Environments

In this paper, we used two different environments to measure the effect of color on eye-hand coordination: Real World (RW) and Virtual Environment (VE) [2_*Environments*_ = (*RW, VE*)]. We kept the lighting conditions and colors similar across both mediums. The details of environments, lighting conditions, and colors are given below:

##### 3.2.1.1 Real world apparatus and color viewing light booth

For this study, we used *Just Normlicht Color Viewing Light 3 BASIC Viewing Booth*, which provides color assessment between D65 (~6,500 K), A (~2,856 K), and TL84 (~4,100 K) illuminants. The dimensions of the color viewing booth were 64 × 33 × 36 cm, and we used all four lights inside the booth to ensure that the color temperature was stable throughout the experiment. The color viewing booth provides a visual assessment of colors according to international standards under constant illumination (Burgos et al., 2014; Nahavandi et al., 2020). In this study, we used this feature of the booth to keep the lighting conditions and colors the same for all participants while they perform a mirror drawing task.

##### 3.2.1.2 Virtual environment

For the virtual environment (VE), software was developed for Oculus Quest 2 and one of its Touch controllers. The software was designed as a standalone application developed on Unity3D (2021 version) with the aid of the Oculus integration package (version 39).

To create the 3D model of the color viewing booth, we tape-measured the booth dimensions and created the same virtual model for VE. By placing virtual controllers on the virtual booth's edges in VE and tape-measuring the distance between them in the real world, we confirmed that its measurement was the same as the RW booth's.

### 3.3 Mirror drawing task

**Figure 1:**
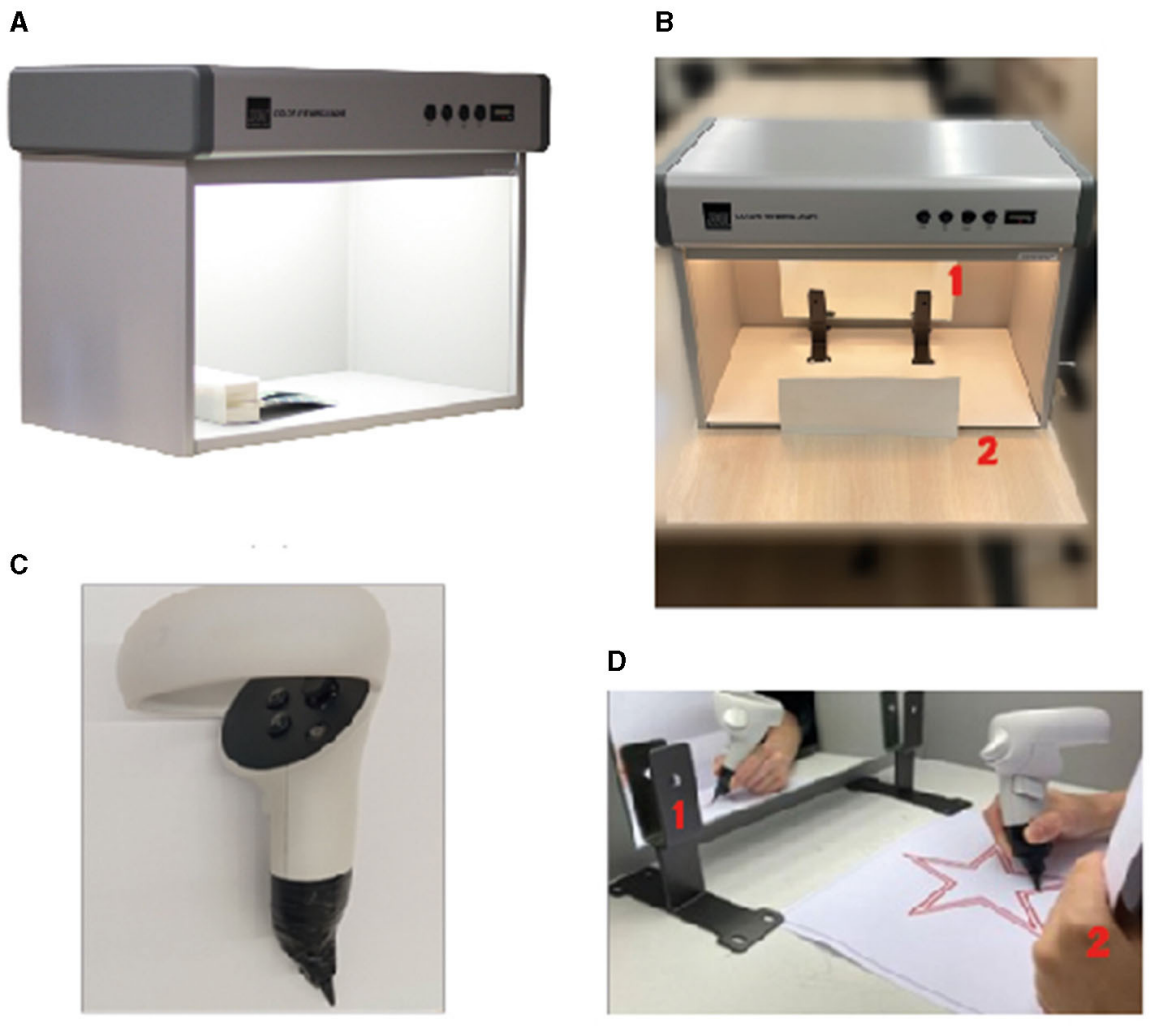
Real world (RW) experimental setup. **(A)** We used a color viewing booth for this study. **(B)** front view of the experimental setup. (1) A mirror placed inside the mirror booth, (2) the participants hold a piece of paper to obscure the view to see the paper in front of them. **(C)** the pencil we used for this study. We attached a pencil to the tip of the Oculus 2 Touch controller and asked participants to trace the start with this input device. **(D)** A participant performing an exemplar trial. (1) The participant looked at the mirror to see their hand and the paper and (2) the participant holding a paper.

For the user study, participants performed a mirror drawing task (Siipola, 1935; Ellis et al., 1957; Marks, 1996; Bhushan et al., 2000), also known as mirror tracing task (O'Boyle and Hoff, 1987; Rouleau et al., 2002; Julius and Adi-Japha, 2016) in both RW and VE. While seated, participants were asked to trace a pentagram shape between two reference lines. One of these lines was the offset of the shape, e.g., the shape is drawn twice with a 0.5 cm offset difference, allowing a distance between the shapes for tracing (Figure 1).

As shown in Figures 1B, D, the shape was placed on the table in front of the participant while the direct line of sight between the participant and the shape was obscured with an object. In this study, we placed a mirror inside the color viewing booth that leaned downwards (Figure 1B). The participants were then asked to look at the tilted mirror that shows a reflection of the star and trace within the offset. The difficulty in the task lies in the hand-eye coordination needed to move when the participant is seeing a reflected view (see Figure 1D).

In the experiment, participants were positioned in front of the booth in VE and RW. The mirror we used was large enough to cover the entire experimental setup where the participants drew the shape (Figures 1B, D). Participants sit on an adjustable stool, where they could adjust the height. By changing the height of the stool, they created an ergonomic environment to perform the task.

**Figure 2:**
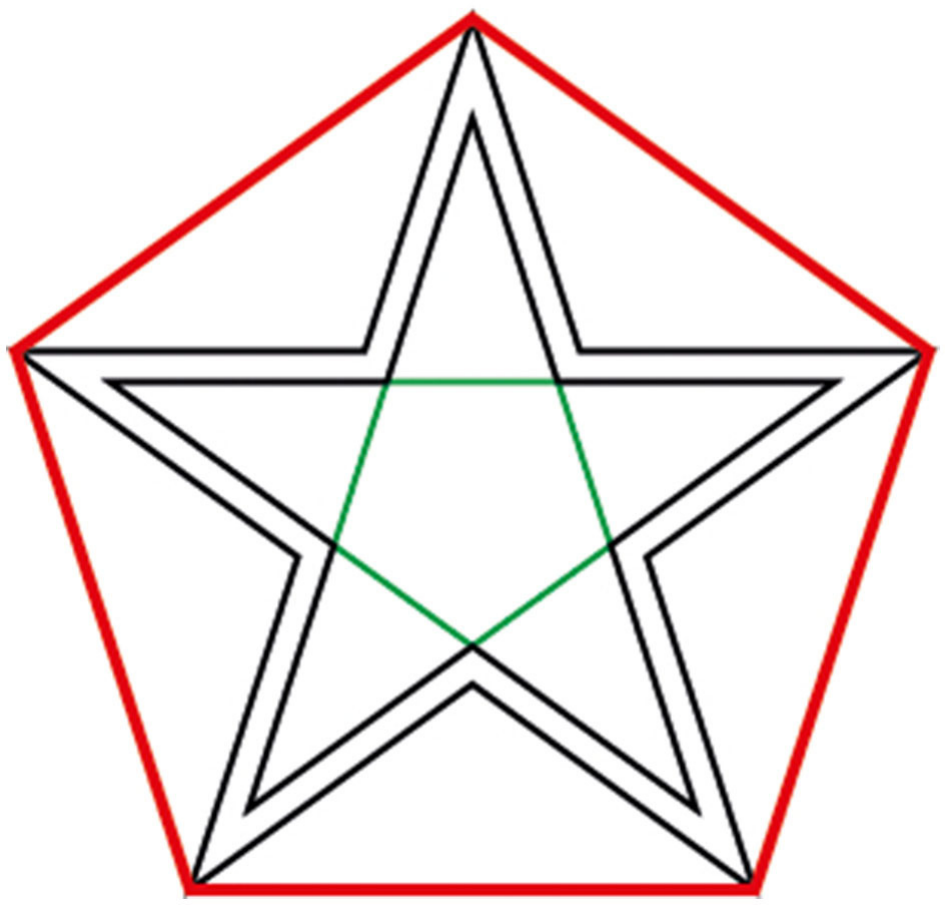
The star used in this study. The participants were asked to trace the start between the lines. The outer red pentagon and interior green pentagon are designed to show that the star shape.

For this study, we used a pentagram (the ratio of inner to outer pentagons defining the star is the golden ratio) (Figure 2). This defines a pentagram that has parallel lines; thus, the number of different directions is five directions. The shape always has a defined starting and ending point. For this study, the starting and ending points were the upper part of the star. The task is considered completed when participants successfully trace the entire shape, starting from the initial point and concluding at the starting point again.

To maintain the same environment and task conditions as the RW, participants performed the study in the VE with the controller inside the color viewing booth. Participants were able to draw shapes on paper with their hands inside the color viewing booth, which was more natural than drawing in mid-air. Both RW and VE experiments were conducted on the same type of paper in the booth, so participants experienced the same haptic feedback and texture.

In other words, the participants performed both experiments in the same area, inside the booth, with the same VR controller apparatus, i.e., input device.

#### 3.3.1 Input device

In this experiment, the same input device was used for both VE and RW. In our pilot studies, we first tried the use of the built-in Oculus HMD hand-tracking algorithm. However, we observed that the hand-tracking performance of the device was not sufficiently accurate for this study. Furthermore, we tried to attach markers to a pen; however, this modified the weight distribution of the pen, and the pilot participants were not comfortable with this approach. We also considered other grip styles, but the most precise interaction method was the precision grip (Batmaz et al., 2020). To accurately track the VR controller in VE, we followed the method in the Kern et al. (2021) study and modified the Oculus Touch controller as seen in Figure 1C.

We used super glue to attach a nut to the lower side of the controller and screwed a short pencil tip into the nut, which provided strength to the apparatus (Kern et al., 2021). Participants performed the experiment with the same controller tip in both RW and VE. Another important reason is to maintain the feeling of the pencil tip that participants had the same haptic perception over the surface of the paper. This also allowed both environments to have the same texture during the drawing, greatly minimizing the differences between the environments. We also recorded participants drawing trajectories in RW and VE by tracking the Oculus Touch controller.

In the VE, the participants saw the controller with the tip of a pen that extends from the bottom of the device to create the same visual image. Moreover, the pencil tips that were cut and screwed were also measured by size according to the tip that extends from the controller in the virtual environment.

#### 3.3.2 Lighting conditions

For both VE and RW, we utilized 6,500 K daylight white to illuminate the task space. In RW, participants were placed in a dark room, and the only source of illumination was the booth. To create the 6,500 K daylight, we used the setting on the color cues booth, which was calibrated by the technician of the company. In VE, we replicated the same lighting conditions and also checked the colors with a spectrophotometer.^1^

#### 3.3.3 Colors and color calibration

**Figure 3:**
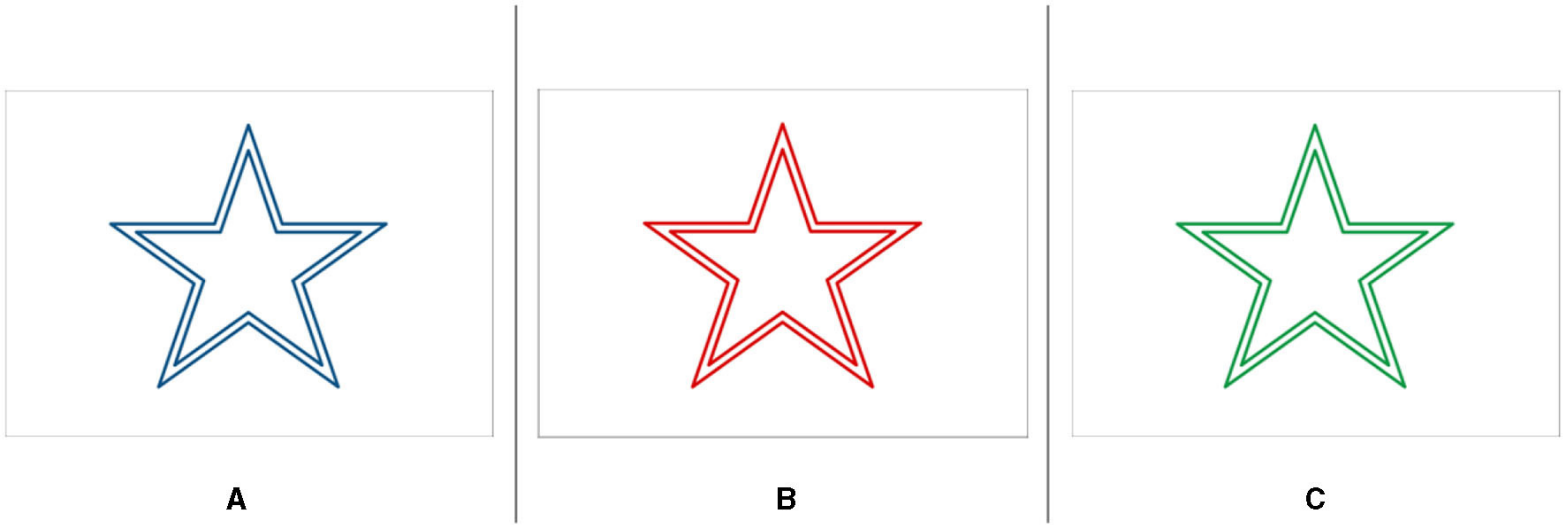
Colors used in this study. **(A)** blue, **(B)** red, and **(C)** green.

In this study, we aimed to use three colors: (3_*color*_ = *red, green, and blue*) and our goal was to create the same perceived colors in both VE and RW. However, using the same digital red, green, and blue color codes in both environments generated different perceived colors because of, e.g., the toner, printer quality, VR headset light, and how colors are rendered in VR. Instead, we first printed red, blue, and green from the same printer, using the following decimal codes in Photoshop (255, 0, 0), (0, 255, 0), and (0, 0, 255), respectively (see Figure 3 for colors). We then measured the colors on the paper and got R = (205, 32, 39), G = (19, 155, 72), and B = (16, 85, 137) with a colorimeter. We used the same color codes in the Unity game engine to create the colors and materials. Then, using a colorimeter, we measured the colors inside the VR HMD and fine-tuned the colors until we got the same color codes as on the paper. It allowed us to generate the same color in both VE and RW.

### 3.4 Procedure

**Figure 4:**
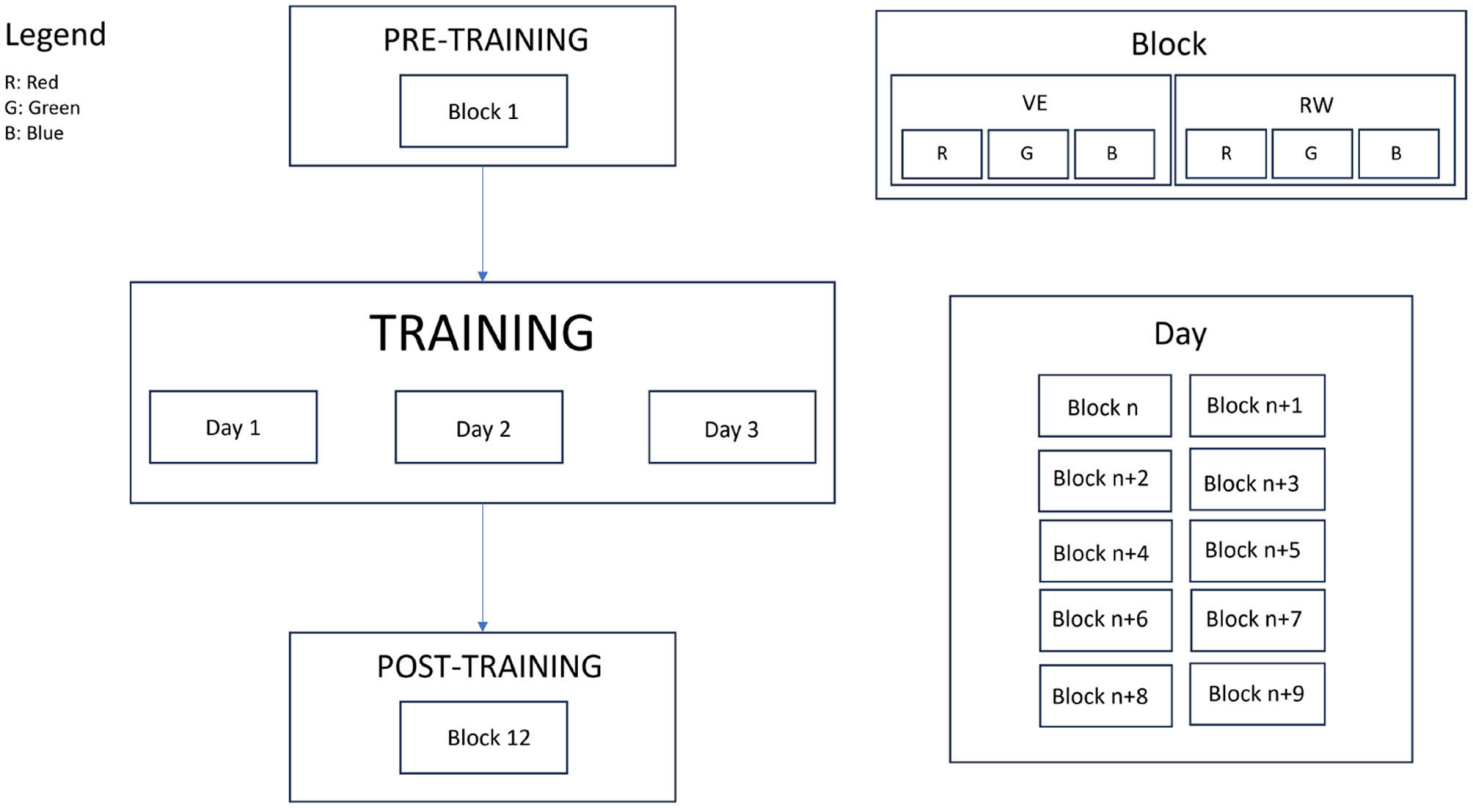
Five days of the experiment. On the first day, participants performed the pre-training session with six trials, which we call in this paper a block. Training sessions lasted for three days, and each day, participants performed 10 blocks. On the last day, participants performed the post-training session with six trials.

In this experiment, we counterbalanced 12 participants with 2_*environment*_ x 3_*colors*_. For training, participants were asked to repeat the experiment for three consecutive days, as shown in Figure 4.

#### 3.4.1 Pre-training session

On the first day, the experimenter explained the experiment with the input device and other tools by introducing the setup. The participants were then asked to sign a consent form and complete the color-blindness test. Before the experiment, participants were asked to fill a demographic questionnaire that included questions about their age, gender, dominant hand, and dominant eye. Afterward, participants were asked to perform one trial from each condition, which was recorded as the pre-experiment results. They were then asked to continue a short survey about their perceived initial performance and preferences according to the pre-training session.

#### 3.4.2 Training sessions

The training sessions lasted 3 days. On each day, participants were asked to trace the star shape in red, green, and blue, both in the RW and in the VE, 10 times. A condition block by completing ten trials of the said block. After that, participants moved to the next block in order until all six blocks were completed. Participants were allowed to take breaks between blocks. When a new block required a change of environment (VE to RW, or visa versa), the experimenter adjusted the equipment and conditions accordingly. The participants completed three consecutive days in the same order. Each training block took around 50 min.

#### 3.4.3 Post training session

On the last day, one trial is collected for each condition. These single trials are the post-experiment data that will be analyzed to improve the data analysis section. On the same day, participants are asked to fill out a post-experiment survey about their perceived performance and opinions on the study.

### 3.5 Experimental setup

**Figure 5:**
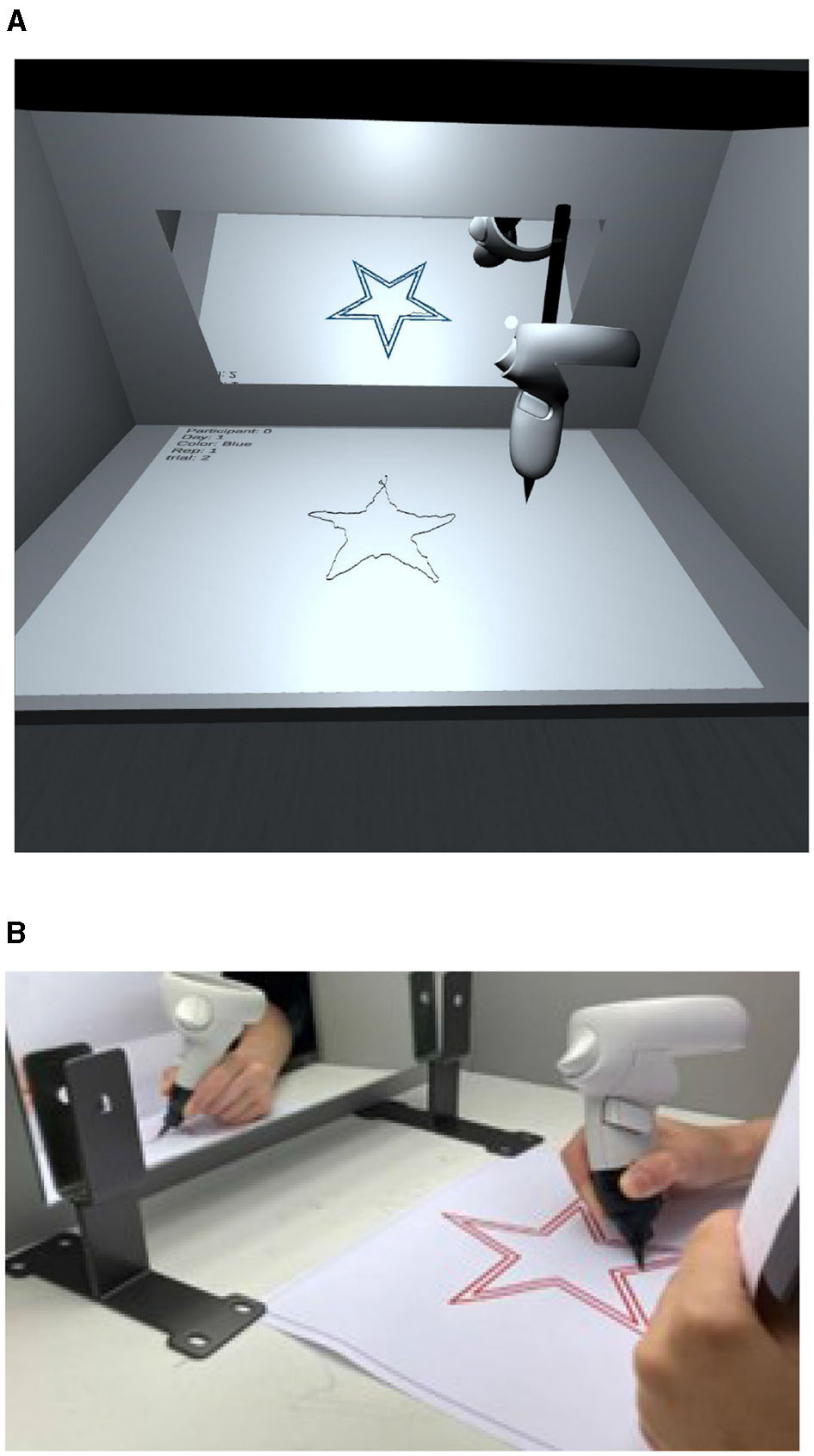
Two different environments used in this paper. **(A)** VR setup in VE. The star shape was not visible to the participants on the paper but on the mirror. **(B)** RW setup. The image on the paper was obstructed by the participant, who held a paper.

Before starting a trial, the experimenter placed a paper in the center of the color viewing booth, as seen in Figure 5. For the VE, the shape color appeared in the correct order automatically according to the participant number. In RW, the experimenter placed the correct color based on the participant number. The experimenter manually calibrated the height of the stool and table to ensure that the surfaces of the physical and virtual color viewing booth were aligned.

When the participants were ready to start a trial, they told the experimenter to start a chronometer. Upon completion of the drawing, the participant notified the experimenter to stop the timer, and the experimenter recorded the elapsed time manually. Afterward, the experimenter swapped the sheet for the next trial paper in RW. To move on to the next trial in the VE, participants pressed a button on the VR controllers, and a new sheet appeared in front of them.

### 3.6 Data collection

For this study, we collected the time and error rate data of the participants. We also calculated the coefficient of variance for time and error rate of the data.

#### 3.6.1 Time

To maintain the same precision in the time data, we measured the task completion time in both VE and RW using a manual chronometer. Participants notified the experimenter when they were going to start and finish the experiment. For the time variable, we measured the duration between these two events in seconds (s).

#### 3.6.2 Error rate

**Figure 6:**
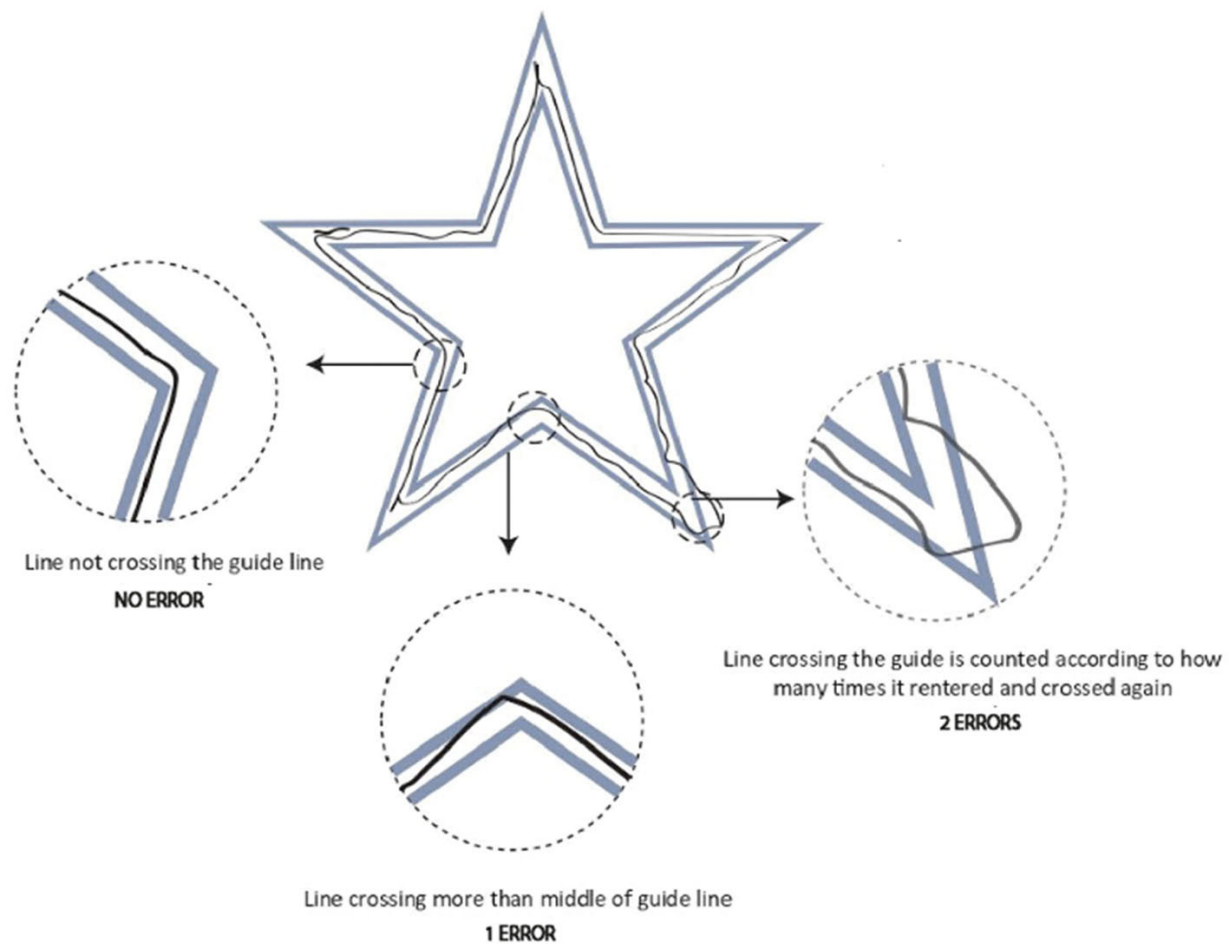
Error calculation method for participant tracing the guide.

We collected data in VE and RW independently to calculate the error rate. After each RW trial, the paper in front of the participant was collected after the experiment, and the experimenter counted the number of errors. In VE, the experimenter pressed the spacebar button on the keyboard to take a top-view screenshot of the trial, and that image was saved to the computer. Then, the experimenter manually counted the number of errors. We defined an “error” as when the stroke of a participant intersects or crosses the midline of the inner or outer guide parallel to the direction of the guide. The guideline for defining an error is shown in Figure 6. The errors were counted by strokes outside the defined boundary, not by how far it was outside the boundary. It is important to note that there were no major outliers in the drawing trajectory where the stroke left the guide for a very long distance. In other words, we did not observe a case where the participants drew a line that left the guide for a long distance and then entered a later (also long) separation from the point of exit.

#### 3.6.3 Coefficients of variation

The coefficient of variation (CV) denotes the proportion of the standard deviation in relation to the mean, providing a measure of the degree of variability relative to the population's mean. A higher CV value indicates a larger dispersion. It is calculated as the ratio of the standard deviation to the mean (Equation 1). In Equation 1, σ shows the standard deviation, and μ shows the population mean.


(1) $$\begin{array}{l} CV=\frac{\sigma }{\mu } \end{array}$$

For training sessions, we measured CV for time and error rate.

### 3.7 Experimental design

The experiment was designed at three levels: pre-, post-, and training. Each participant performed the experiment in two different environments (2_*Environment*_: RW and VE), and for the three different colors (3_*Color*_: red, blue, and green). We collected the task execution times and number of errors of the participants and calculated CV.

In the pre-training session, participants performed the 2_*Environment*_ × 3_*Color*_ conditions. In the training session, the participants repeated the same task 10 times, yielding 2_*Environment*_ × 3_*Color*_ × 10_*repetitions*_. In the post-experiment session, as in the pre-experiment session, each participant performed one trial for 2_*Environment*_ × 3_*Color*_ conditions. In total, a participant performed 2_*Environment*_ × 3_*Color*_ × 2 days + 2_*Environment*_ × 3_*Color*_ × 10_*repetitions*_ × 3 days = 192 trials.

## 4 Results

We used Microsoft Excel to record the data of the participants, such as participant number, age, dominant hand, dominant eye, case, number of errors, and time. The data were then transferred, distributed, and tabulated after the experiment was completed for all participants to JMP 16 in order to plot the graphs. To analyze the results, we used Repeated Measures (RM) ANOVA in SPSS 24. We considered the data to be normal when the Skewness (S) and Kurtosis (K) of the data distribution were within ±1 (Mallery and George, 2003; Hair et al., 2014). Otherwise, we used log-transform before ANOVA. If the data was not normally distributed after the log-transform, we used ART (Wobbrock et al., 2011) before ANOVA. We used the Bonferroni method for *post-hoc* analyses and applied Huynh-Feldt correction when ε < 0.75 if sphericity was violated. The graphs shown in the figures show the mean, and the error bars represent the standard error of the mean. We first analyzed the general results, then the pre- and post-training results, and finally the training results separately. All the trial data is given in Supplementary material.

### 4.1 General results

**Figure 7:**
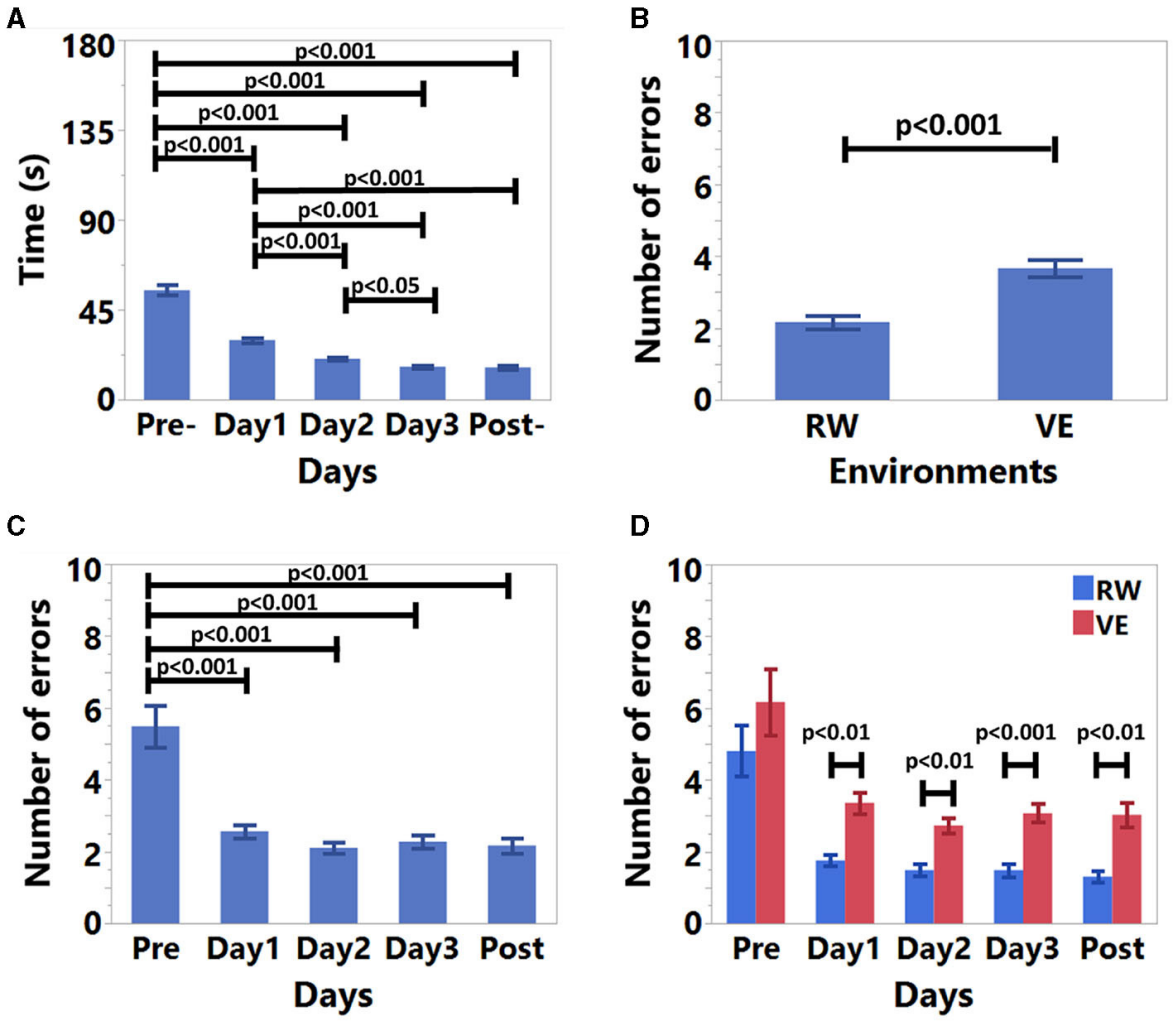
Experiment results. **(A)** Time results for days, **(B)** average number of error results for environments, **(C)** average number of error results for days, and **(D)** average number of error results for days and environments interaction.

For the training results, the time (*S* = 0.39, *K* = −0.41) and error rate (*S* = 0.22, *K* = 0.18) data had a normal distribution after log-transformation. The results are shown in Table 1 and Figure 7. According to Figures 7A, C, participants were slower, and they made more errors in the pre-training session. Figure 7B results indicate that participants made more errors in VE. The significant interaction results between days and environments in Figure 7D shows that except for pre-training, participants made fewer errors in RW.

**Table 1:** Training session results for environment, color, and day.

	**Environment**	**Color**	**Day**	**Environment × color**	**Environment × day**	**Color × day**	**Day × board × feedback**
Time	*F*_(1,11)_ = 0.932, *p* = 0.355, η^2^ = 0.078	*F*_(2,22)_ = 2.46, *p* = 0.109, η^2^ = 0.183	***F*_(1.73, 19.07)_ = 94.56,** ***p* < 0.001,** **η^2^ = 0.896**	*F*_(2,22)_ = 0.871, *p* = 0.416, η^2^ = 0.073	*F*_(4,44)_ = 0.397, *p* = 0.810, η^2^ = 0.035	*F*_(8,88)_ = 0.901, *p* = 0.520, η^2^ = 0.076	*F*_(8,88)_ = 0.475, *p* = 0.871, η^2^ = 0.041
Error rate	***F*_(1,11)_ = 16.206,** ***p* < 0.01,** **η^2^ = 0.596**	***F*_(2,22)_ = 1.16,** ***p* = 0.222,** **η^2^ = 0.128**	*F*_(1.77, 19.52)_ = 4.688, *p* < 0.01, η^2^ = 0.299	*F*_(2,22)_ = 0.378, *p* = 0.689, η^2^ = 0.033	***F*_(4,44)_ = 2.717,** ***p* < 0.05,** **η^2^ = 0.198**	*F*_(8,88)_ = 1.991, *p*=0.056, η^2^ = 0.154	*F*_(8,88)_ = 1.349, *p* = 0.32, η^2^ = 0.109

Significant results are shown in bold.

### 4.2 Pre and post training results

**Figure 8:**
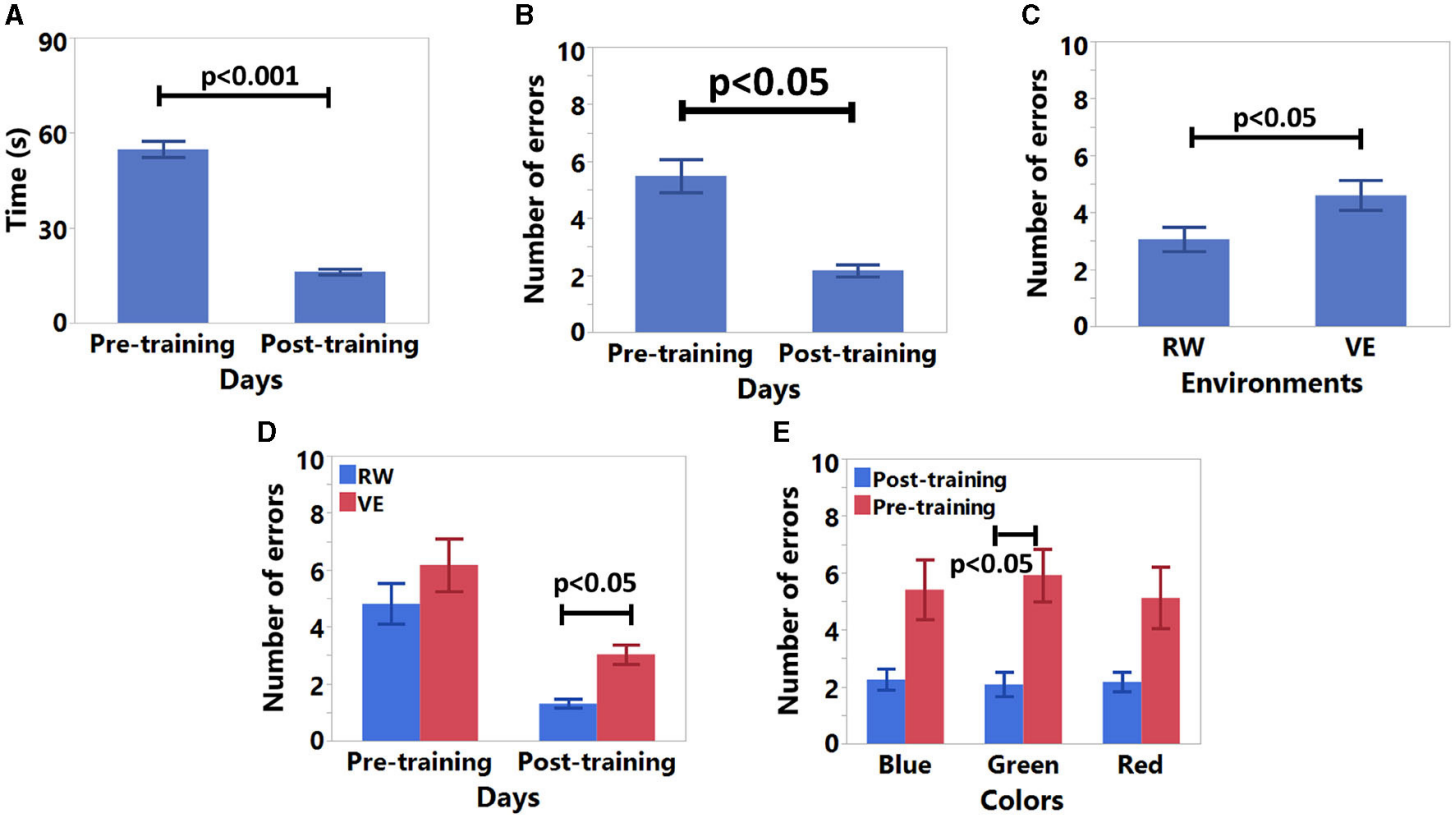
Pre- and post-training data results. **(A)** Time results for days, **(B)** average number of errors results for days, **(C)** average number of errors results for environment, **(D)** average number of errors results for days and environment conditions, and **(E)** average number of errors results for days and colors conditions.

For pre and post-training results, the error rate exhibits normal distribution after log-transformation (*S* = −0.06, *K* = −0.54). Time data did not exhibit a normal distribution after log-transformation, so we applied ART before RM ANOVA. The results are shown in Table 2 and Figure 8. According to these results, participants were faster (Figure 8A) and made fewer errors (Figure 8B) in post-training. Furthermore, participants made more errors in VE (Figure 8C). The significant interaction result on days and environments conditions showed that participants made fewer errors in RW in the post-training session (Figure 8D). Finally, we found a significant interaction result between sessions and colors. According to these results, participants made fewer errors with green color in post-training (Figure 8E).

**Table 2:** Pre- and post-training session results for environment, color, and day.

	**Environment**	**Color**	**Day**	**Environment × color**	**Environment × day**	**Color × day**	**Day × board × feedback**
Time	*F*_(1,11)_ = 0.175, *p* = 0.684, η^2^ = 0.016	*F*_(2,22)_ = 0.69, *p* = 0.512, η^2^ = 0.059	***F*_(1,11)_** **= 209.36**, ***p***** < 0.001**, **η^2^** **= 0.95**	*F*_(2,22)_ = 1.724, *p* = 0.202, η^2^ = 0.135	*F*_(1,11)_ = 0.637, *p* = 0.442, η^2^ = 0.055	*F*_(2,22)_ = 0.012, *p* = 0.988, η^2^ = 0.001	*F*_(2,22)_ = 0.481, *p* = 0.625, η^2^ = 0.042
Error rate	***F*_(1,11)_** **= 5.06**, ***p***** < 0.05**, **η^2^** **= 0.315**	*F*_(2,22)_ = 0.712, *p* = 0.501, η^2^ = 0.061	***F*_(1,11)_** **= 8.795**, ***p***** < 0.05**, **η^2^** **= 0.444**	*F*_(2,22)_ = 0.683, *p* = 0.515, η^2^ = 0.058	***F*_(1,11)_** **= 7.665**, ***p***** < 0.05**, **η^2^** **= 0.411**	***F*_(2,22)_** **= 3.61**, ***p***** < 0.05**, **η^2^** **= 0.191**	*F*_(2,22)_ = 1.248, *p* = 0.307, η^2^ = 0.102

Significant results are shown in bold.

### 4.3 Training results

**Figure 9:**
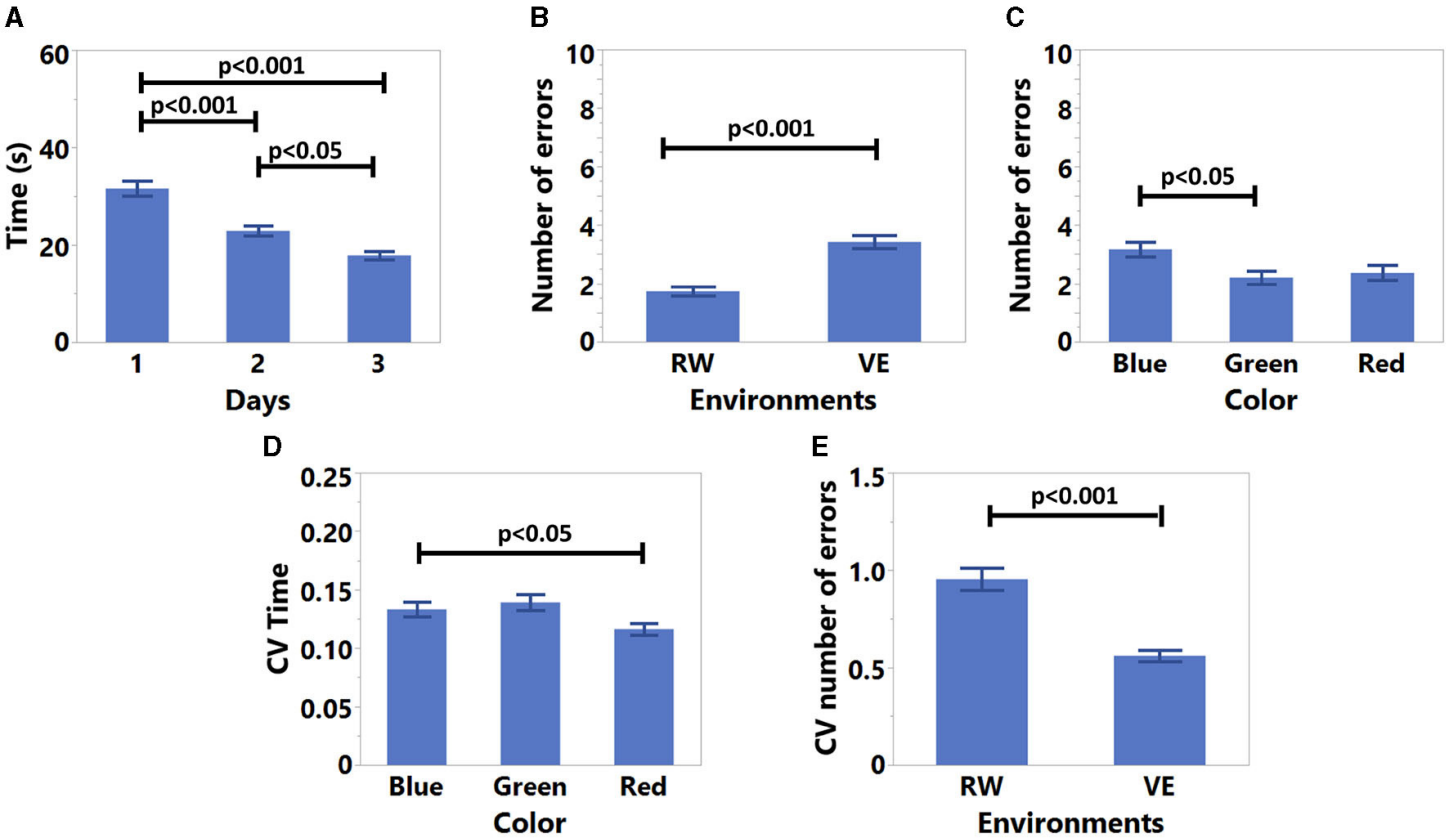
Three days training results. **(A)** Time results for days, **(B)** average number of errors results for environments, **(C)** average number of errors results for colors, **(D)** CV time results for colors, and **(E)** CV number of errors results for environments.

For the training results, the error rate exhibited a normal distribution (*S* = 0.87, *K* = 0.68). Time (*S* = 0.004, *K* = −0.37) and CV Time (*S* = −0.003, *K* = −0.14) were normally distributed after log-transformation. For CV number of errors, we used ART before ANOVA. The results are shown in Table 3 and Figure 9. According to these results, participants were significantly getting faster each day (Figure 9A). Moreover, participants made more errors in VE (Figure 9B), and with blue color compared to green (Figure 9C). According to Figure 9D, participants exhibited higher CV time results with Blue compared to Red, and the CV number of errors was lower in VE (Figure 9E).

**Table 3:** Three-day training session results for time, error rate, CV time, and CV error rate.

	**Environment**	**Color**	**Day**	**Environment × color**	**Environment × day**	**Color × day**	**Environment × color × day**
Time	*F*_(1,11)_ = 0.153, p = 0.703, η^2^ = 0.014	*F*_(2,22)_ = 1.646, p = 0.216, η^2^ = 0.130	***F*_(1.32, 14.57)_ = 23.749,** **p < 0.001,** **η^2^ = 0.683**	*F*_(2,22)_ = 1.451, p = 0.256, η^2^ = 0.117	*F*_(2,22)_ = 0.574, p = 0.571, η^2^ = 0.05	*F*_(4,44)_ = 2.301, p = 0.074, η^2^ = 0.173	*F*_(4,44)_ = 0.611, p = 0.657, η^2^ = 0.053
Error rate	***F*_(1,11)_ = 33.071,** **p < 0.001,** **η^2^ = 0.75**	**_*F*(1.24, 13.68)_ = 5.563,** **p < 0.05,** **η^2^ = 0.336**	*F*_(2,22)_ = 1.374, p = 0.274, η^2^ = 0.111	*F*_(2,22)_ = 1.634, p = 0.218, η^2^ = 0.129	*F*_(2,22)_ = 0.211, p = 0.811, η^2^ = 0.019	*F*_(4,44)_ = 1.185, p = 0.331, η^2^ = 0.097	*F*_(4,44)_ = 0.770, p = 0.550, η^2^ = 0.065
**CV Time**	*F*_(1,11)_ = 0.778, p = 0.397, η^2^ = 0.066	***F*_(2,22)_ = 3.39,** **p < 0.05,** **η^2^ = 0.236**	*F*_(2,22)_ = 0.47, p < 0.954, η^2^ = 0.004	*F*_(2,22)_ = 0.203, p = 0.818, η^2^ = 0.018	*F*_(2,22)_ = 0.519, p = 0.602, η^2^ = 0.045	*F*_(4,44)_ = 0.767, p = 0.552, η^2^ = 0.065	*F*_(4,44)_ = 1.157, p = 0.343, η^2^ = 0.095
**CV** **Error rate**	***F*_(1,11)_ = 29.004,** **p < 0.001,** **η^2^ = 0.725**	*F*_(2,22)_ = 0.258, p = 0.775, η^2^ = 0.023	*F*_(2,22)_ = 0.187, p = 0.830, η^2^ = 0.017	*F*_(2,22)_ = 1.233, p = 0.331, η^2^ = 0.101	*F*_(2,22)_ = 0.054, p = 0.948, η^2^ = 0.005	*F*_(4,44)_ = 1.525, p = 0.211, η^2^ = 0.122	*F*_(4,44)_ = 1.953, p = 0.119, η^2^ = 0.151

Significant results are shown in bold.

## 5 Discussion

In this paper, we conducted a 5-day user study with 12 participants in RW and VE, where they performed a mirror drawing task with three different colors. Our objective was to understand how different colors affect user motor performance in a motor learning task between RW and VE.

### 5.1 General results

Overall, the results of the experiment show that the participants were getting faster and making fewer errors with a mirror drawing task after 5-days of training. We did not find any statistically significant interaction results between the last day of training and the post-training in terms of time and number of errors, indicating that the learning curvature of the participants reached a plateau. In other words, we have collected sufficient sample data to represent the learning curve of the participants. Furthermore, when we looked at the individual training results, we did not observe any large deviations for time and number of errors. Moreover, our results exhibit a large effect size η^2^ > 0.14, which indicates a strong effect. Therefore, even though we collected data from 12 participants, we believe that our findings are robust and expect that when the experiment is replicated, the probability of observing the same results is high.

In this experiment, we asked participants to draw the star shape as fast and as precise as possible, i.e., we did not ask them to prioritize any of the task execution strategies. According to the results, while we found a decrease in the task execution time, we only observed a significant difference in the number of errors between the pre-training and subsequent days. This result indicates that the motor performance of the participants improved in terms of time, but not in terms of the number of errors during training sessions. We believe that this is an outcome of the mirror-drawing task and how the participants learned it (Allen, 1948). For future studies, we also recommend including different task execution strategies to analyze how user motor learning is affected in terms of time and number of errors.

In addition, there may be individual differences in the way individuals respond to RW and VE training environments, and other factors such as individual skill level, motivation, adaptation time, and task complexity may also play a role in determining performance and learning outcomes.

### 5.2 Training across realities: RW vs. VE

The results of our study showed that participants made more errors in VE during the entire experiment. Based on previous literature, this result was expected. The limitations of existing VR HMDs, such as stereodeficiencies (Batmaz et al., 2022), imperfect visual depth cues (Ragan et al., 2009), or other visual perception-related issues (see Lin and Woldegiorgis, 2015 for a review), might have an impact on sensory-based motor performance and detrimental effects on user performance. Although we kept both environments as similar as possible, we speculate that this increase in errors is related to the limitations of the VR HMD systems.

As one of the hardware limitations, the tracking precision of the VR controller may also contribute to these results. The previous studies have shown that Oculus Quest 2 can achieve a sub-mm accuracy, e.g., 0.06 mm (Holzwarth et al., 2021). We conducted the experiment in a dark room with no external light sources, and the only light source was light from the color viewing booth, so we do not anticipate adverse effects of the illumination on the tracking accuracy. Furthermore, the experimenter did not observe any sudden changes in tracking precision that might affect the results. Even so, we acknowledge that the virtual controller we rendered was completely synthetic, which means the visual feedback received by the participants might be affected by the jitter in tracking accuracy.

When we looked at the detailed analysis of the number of errors, we also observed that there was no significant difference in pre-training between RW and VE. Since we counterbalanced the experiment with a Latin square and used a within-subjects design, we believe that this was not caused by bias in the data. However, we speculate that this result is an outcome of the cognitive load of the participants during learning. Based on the individual time and error data in the Supplementary material, it can be seen that participants had difficulty completing the task for the first time, similar to previous mirror drawing task studies, e.g., (Allen, 1948; Julius and Adi-Japha, 2016; Liu et al., 2021). The participants experienced a higher cognitive load during this learning period (Allen, 1948). Moreover, as we did not observe any significant differences in time between RW and VR in pre-training, we speculate that the detrimental effects of VR HMD limitations or controller tracking did not become apparent at longer task execution times. In other words, as the participants learned the task and became faster, we began to observe the limitations of the VR HMD on a deeper level. All of these speculations, however, require further investigation.

Although there is a significant difference between RW and VE, our results show that VR HMDs still provide a platform to perform mirror-drawing tasks. The learning curve in VE and the decrease in the number of errors suggest that individual motor performance can be improved with practice in virtual reality, even in tasks that are not associated with traditional motor learning methods.

### 5.3 The effect of colors on motor performance

In this experiment, we did not observe any significant interaction between colors and environments for time and number of errors. Similarly, CV results also did not reveal a significant interaction. We believe that our method of generating the same colors across both VE and RW provided equivalent visual stimuli as we kept them as similar as possible. Our results suggest that the tested colors are not sensitive to environments. This indicates that the differences between the two environments are probably not related to the color change in visual stimuli.

One of the limitations of our study was that we were unable to create primary color codes in red (255, 0, 0), green (0, 255, 0), and blue (0, 0, 255) in real life. The type of paper, the shape that is used, the quality of the ink, the printer that is used, etc., might vary the colors, and how they are printed on the paper. We also considered other options, such as using a tablet to create these colors; however, in this case, participants would be drawing on a tablet surface with a different haptic feedback. Also, they would receive synthetic 2D visual feedback. Thus, we decided that using paper was the best option, as it would more closely mimic the haptic and visual feedback that participants would receive in a real task.

Even though we did not observe a significant interaction between colors and environments, we found significant differences in participants' performance with different colors. Participants made fewer errors with green in post-training. Similarly, the number of errors was significantly fewer with green than with blue during 3-day training. Green is associated with harmony, tranquility, and peace in some cultures (Eren et al., 2022). When people see the color green, it can evoke positive emotions and feelings of relaxation (Kaya and Epps, 2004). This relaxed state of mind can lead to better concentration and focus, reducing the likelihood of making errors due to distractions or stress. Moreover, green is located in the middle of the visible light spectrum, making it easier on the eyes compared to colors at the extreme ends of the spectrum, such as red or blue (Wegman and Said, 2011). Prolonged exposure to colors such as red can cause eye strain, which may contribute to errors. Green, on the other hand, is soothing to the eyes and can help maintain visual comfort, reducing the chances of misinterpreting or omitting information. Moreover, the color green is associated with environments where fewer errors are tolerated or where precision is crucial. For example, in healthcare settings, surgical instruments and hospital attire are often green, which may create an association with accuracy and precision (Herman et al., 2005; Ćurĉić et al., 2019). We believe that we observed a similar color effect on the participants' psychology and, thus, on their motor performance.

We also found that participants had a lower CV in time with red compared to blue. This result indicates that the data points for the red were less spread and more tightly clustered around the mean compared to the blue condition, e.g., the data for the red had less dispersion relative to the mean compared to the data for the blue condition. Red is often associated with alertness and attention (Wegman and Said, 2011). It can stimulate the central nervous system and increase alertness (Figueiro et al., 2009). Participants may have been more focused and attentive when they received the color red as visual feedback, leading to more consistent and precise response times, resulting in a lower CV in time.

In short, the impact of color on motor performance can be a combination of psychological, physiological, and environmental factors. Colors evoke specific psychological associations, influencing mood and arousal levels, which, in return, can affect motor performance (Elliot and Maier, 2007; Hulshof, 2013). Attention and visibility are crucial components, with bright and vibrant colors often enhancing focus, while softer tones reduce visual strain (Elliot et al., 2007). The spatial perception and contrast between colors can further influence how individuals plan and execute motor tasks (Glover, 2004).

The area of visual cues presented in this study was confined. Since participants had to draw a shape between two lines, the thicknesses of both shapes were limited, creating a restricted surface for different colors. This also limits the stimuli and the amount of visual feedback that we can provide to the participants. However, this limitation comes from the nature of the task; that is, participants had to draw the shape within a gap determined by two borders. On the other hand, previous studies showed that the participant's gaze focus on just where they are tracing (Gowen and Miall, 2006; Tchalenko, 2007; Coen-Cagli et al., 2009; Türkmen et al., 2022). Tchalenko (2007) found that in eye-pursuit behavior, the user closely follows their hand with their eyes. Users also use specific eye-scan paths, where they focus only on the parts of the object they are drawing and follow a scan path that resembles an edge-following pattern along image contours (Coen-Cagli et al., 2009). Additionally, tracing requires constant comparison between the line to be traced and the pen tip (Gowen and Miall, 2006; Türkmen et al., 2022). All these previous studies indicate that the participants were looking at and focused on the gap between two lines, i.e., where they were drawing, and we changed the color of the lines to provide visual feedback. Thus, we think that visual feedback with different color cues affected user performance, but not differently across realities.

Although we did not find a significant difference in eye-hand coordination training with a mirror-drawing task between VE and RW for different colors, we recommend future researchers investigate further the effect of color on human perception and emotion, and how it can affect motor learning of users in VE and RW for different fields and setups. The use of color in VR environments may lead to stronger emotional responses and greater levels of engagement from users, which may, in turn, influence their behavior and decision-making processes. On the contrary, the effect of color in real-life experiments may be more subtle or influenced by a wider range of factors, such as cultural context, personal experiences, and individual preferences. Various psychological factors, biological factors, and cultural contexts contribute to our reactions to colors (Renaud and Blondin, 1997). In that regard, it is critical to grasp the psychological impacts that colors may have on humans, as well as the principles of color theory and color meanings.

Our findings have practical applications in various real-world and virtual-world scenarios. In tasks demanding precision and minimizing errors, we recommend considering using colors associated with calmness, such as green, to enhance concentration and focus. For instance, the tasks that demand focus and accuracy, incorporating calming colors could potentially contribute to a more productive work environment. In contrast, for activities that require increased attention, such as certain rehabilitation exercises, the use of alert colors such as red might be beneficial. These findings are also aligned with the previous work (Elliot et al., 2007; Figueiro et al., 2009) and color theory (Wegman and Said, 2011). Similarly, in VR environments, where immersion is crucial, understanding the impact of color on user psychology can guide the selection of color schemes. By choosing colors that evoke positive emotions and relaxation, VR experiences can be designed to be more engaging and enjoyable, potentially leading to increased participation and adherence in training or therapeutic activities. Moreover, in digital interfaces involving motor tasks, such as drawing or design applications, we suggest considering the psychological and physiological impact of colors. Tailoring color schemes for specific goals can enhance the focus of trainees and reduce errors for precision-oriented tasks.

## 6 Conclusion

In this paper, we examined the user motor performance by means of eye-hand coordination in mirror drawing for three days in Virtual Environment (VE) and Real World (RW), with three different colors. Our results showed that participants made fewer errors in RW compared to VR, indicating that the hardware and software limitations of Virtual Reality (VR) Head Mounted Displays (HMDs) have detrimental effects on user motor performance. However, we did not observe significant interaction results in the pre-training session. This can be explained by the high cognitive load on the first training day, potentially leading to variations in motor learning of the participants across different realities. Furthermore, the participants showed progress in terms of time, but not in terms of the number of errors during the training sessions, indicating that they prioritized task execution time even without explicit instructions to focus on time.

As for colors, the results highlight an important outcome for green. Participants consistently exhibited fewer errors when tasked with mirror drawing in this hue. This preference can be attributed to the well-established associations of green with harmony, tranquility, and peace in color theory. Additionally, given its position in the middle of the visual spectrum, green emerges as an optimal choice for tasks requiring sustained visual attention, as it effectively moderates visual fatigue. Moreover, since we did not observe any significant interaction results between the colors and environment conditions, it suggests that participants' motor learning performance is likely to follow similar learning curves in both RW and VR when the same colors are used. This understanding of color effects on motor performance offers practical implications for designing user-friendly and visually optimized environments across a variety of applications.

Overall, our results show that VR systems have the potential to be used as a motor-learning testbench in future mirror-drawing tasks to evaluate participants' eye-hand coordination. A better understanding of the relationship between eye and hand movements in VE can help design more intuitive user interfaces for various applications.

We hope that our results can contribute to various disciplines in which eye-hand coordination plays a critical role in the success of the task, such as medicine and surgery, density, arts & fine arts, interior architecture, industrial design, sports, e-games, and structural engineering. Our findings could be integrated into existing and future RW and VE training applications to enhance their efficacy while keeping user-friendliness in mind. In addition, further research in fields such as psychology, design, and human-computer interaction can help refine and expand the practical applications of these findings in real-life scenarios.

## Data availability statement

The datasets presented in this study can be found in online repositories. The names of the repository/repositories and accession number(s) can be found below: https://osf.io/smcr4/.

## Ethics statement

The studies involving humans were approved by Kadir Has University, Ethics Board Committee. The studies were conducted in accordance with the local legislation and institutional requirements. The participants provided their written informed consent to participate in this study. Written informed consent was obtained from the individual(s) for the publication of any potentially identifiable images or data included in this article.

## Author contributions

ZA: Conceptualization, Data curation, Methodology, Resources, Visualization, Writing—original draft, Writing—review & editing. MHM: Data curation, Investigation, Methodology, Software, Writing—original draft, Writing—review & editing. BM: Conceptualization, Data curation, Formal analysis, Funding acquisition, Investigation, Methodology, Project administration, Resources, Supervision, Writing—original draft, Writing—review & editing. AUB: Conceptualization, Data curation, Formal analysis, Methodology, Project administration, Resources, Supervision, Validation, Visualization, Writing—original draft, Writing—review & editing.

## Footnotes

^1^Konica Minolta spectrophotometer CM-26d.
