# Supplementary material for: Effects of color cues on eye-hand coordination training with a mirror drawing task in virtual environment
Source: Front Psychol. 2024 Jan 15;14:1307590. doi: 10.3389/fpsyg.2023.1307590 (PMC10823539; doi:10.3389/fpsyg.2023.1307590)
Supplement: Supplementary file 1 [file Data_Sheet_1.PDF]

Supplementary Material

1 SUPPLEMENTARY TABLES AND FIGURES

1.1 Figures

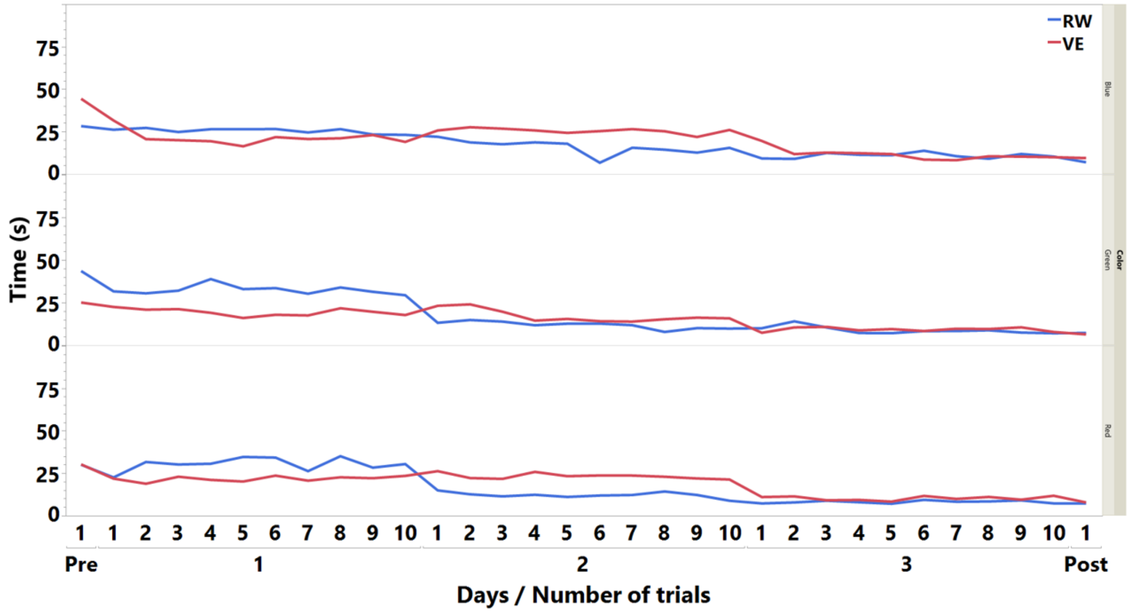

Figure S1. Participant 1 Time Results

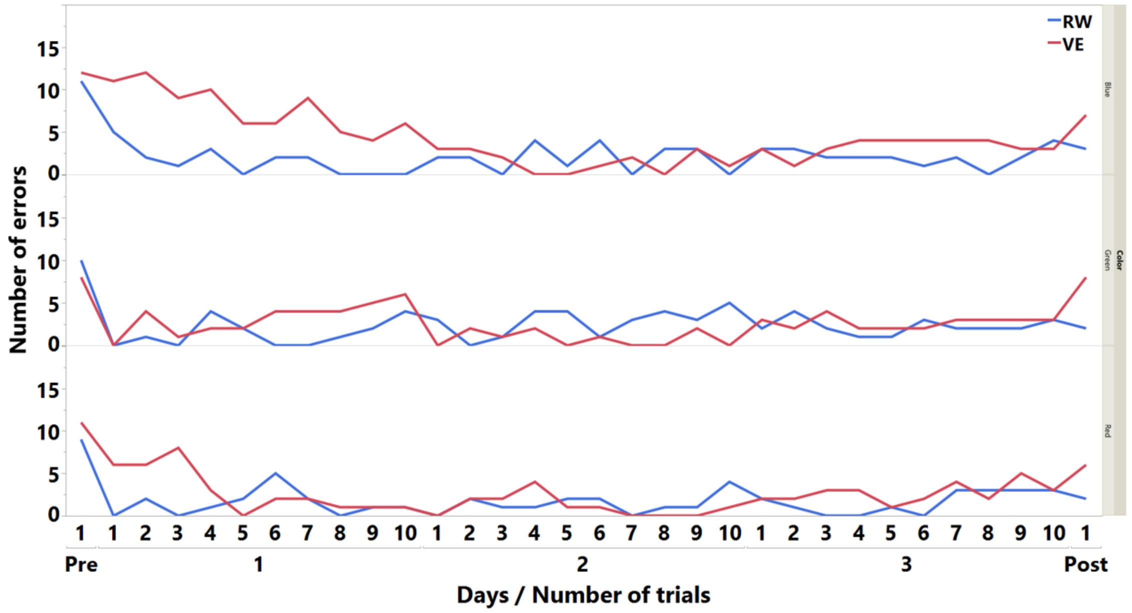

Figure S2. Participant 1 Number of Errors Results

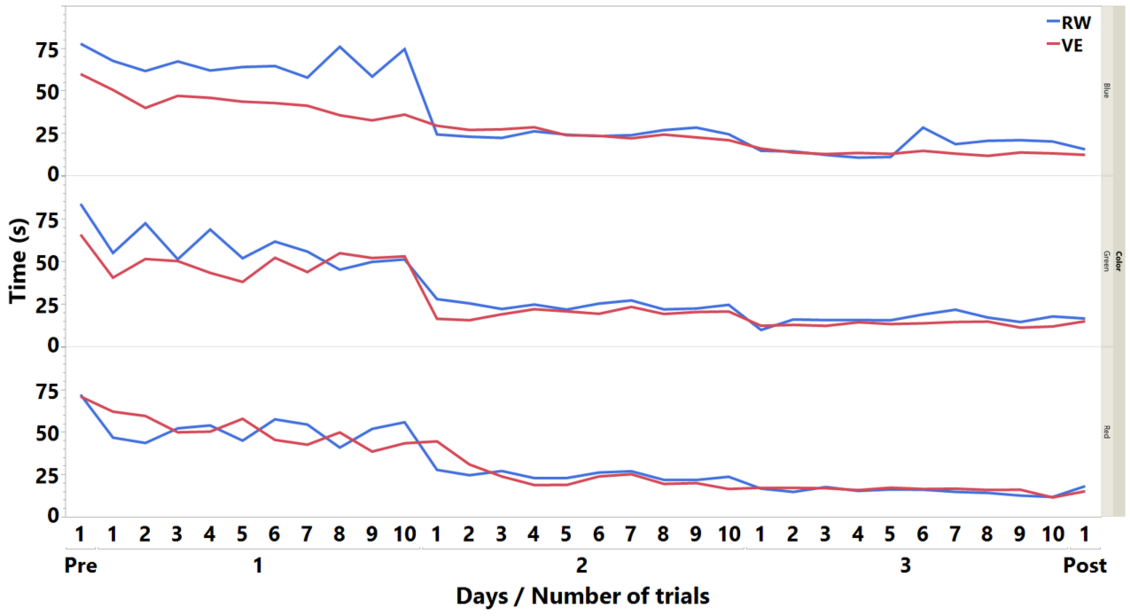

Figure S3. Participant 2 Time Results

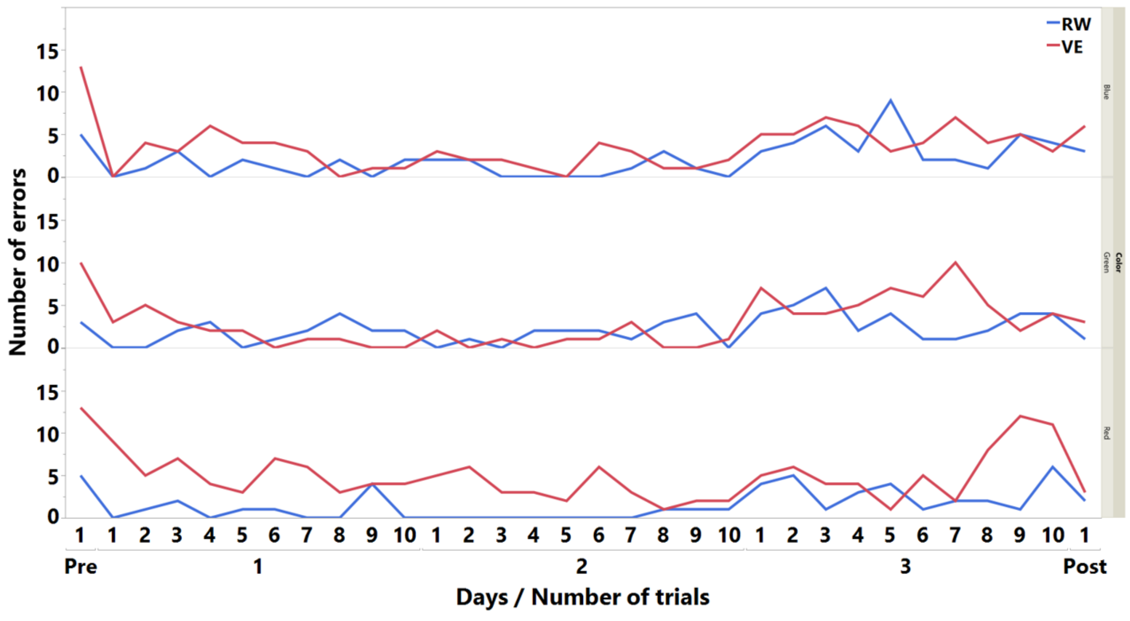

Figure S4. Participant 2 Number of Errors Results

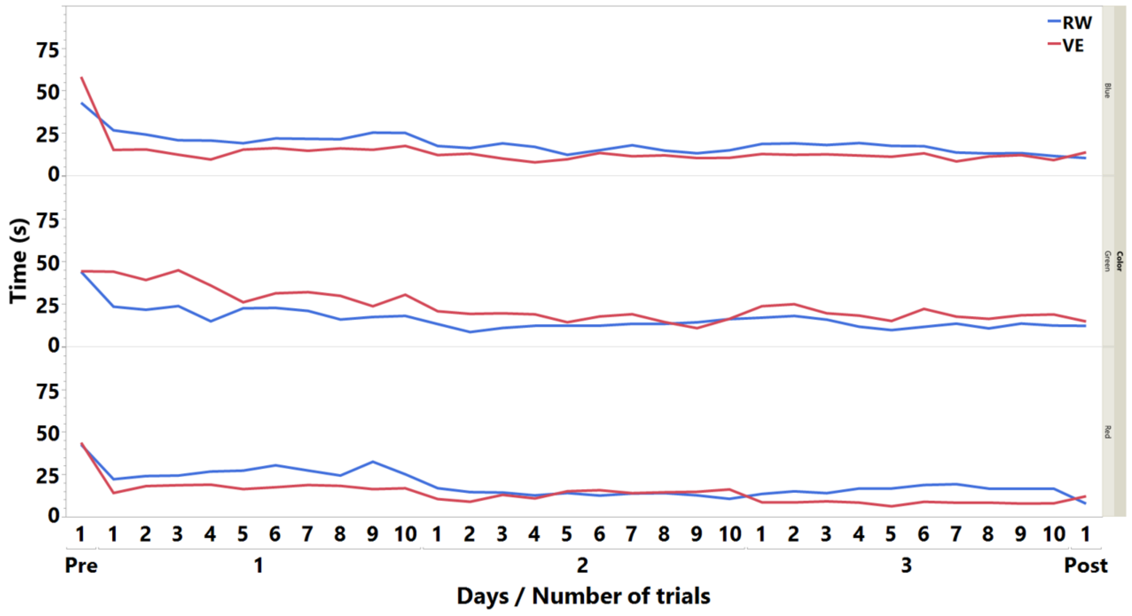

Figure S5. Participant 3 Time Results

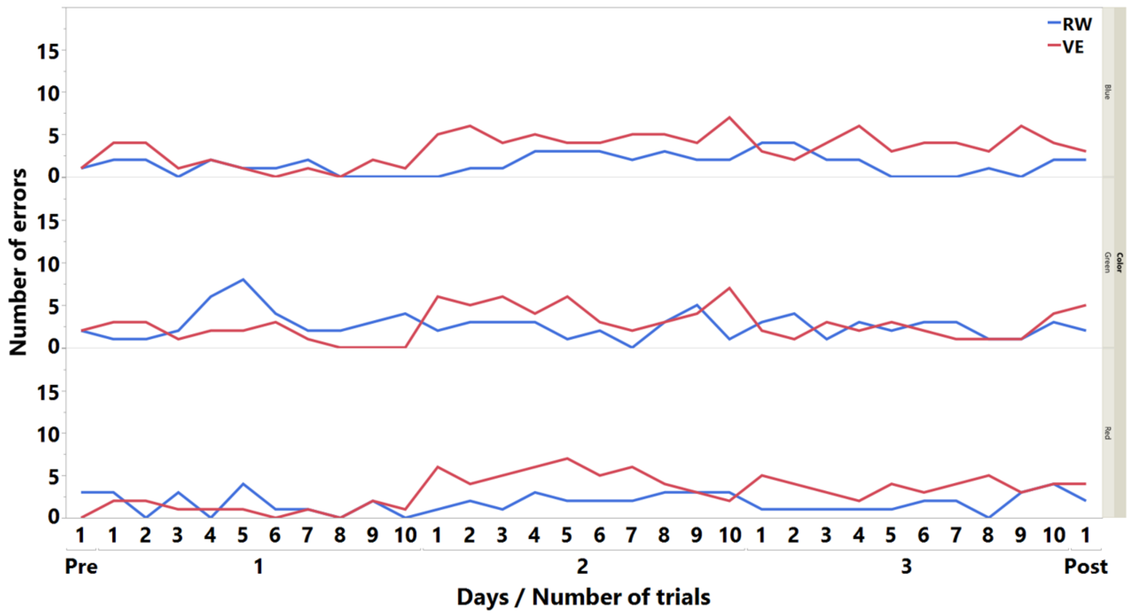

Figure S6. Participant 3 Number of Errors Results

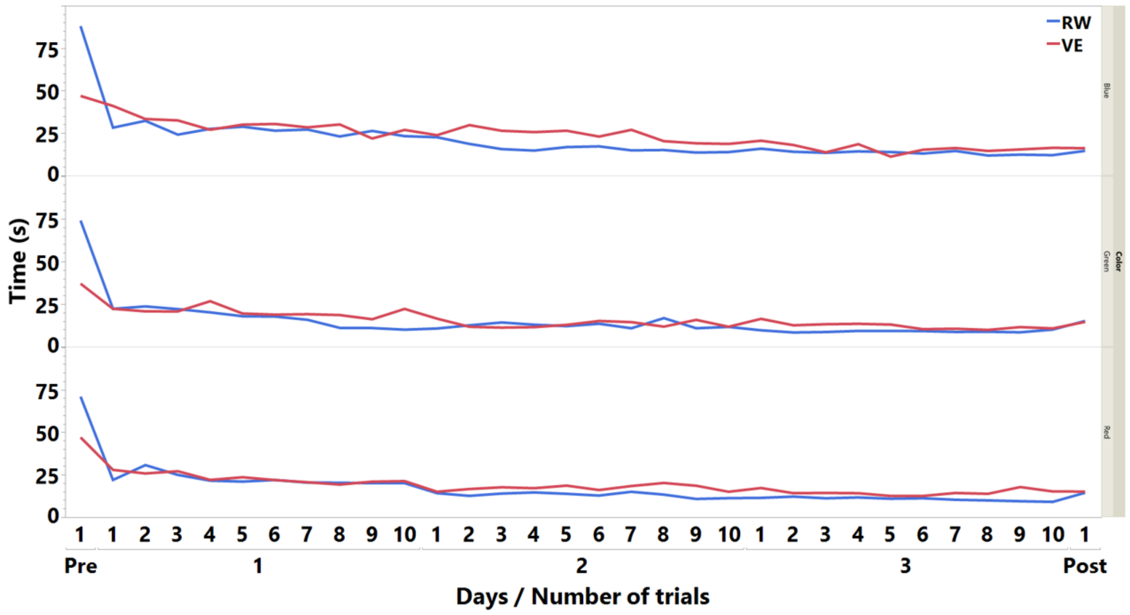

Figure S7. Participant 4 Time Results

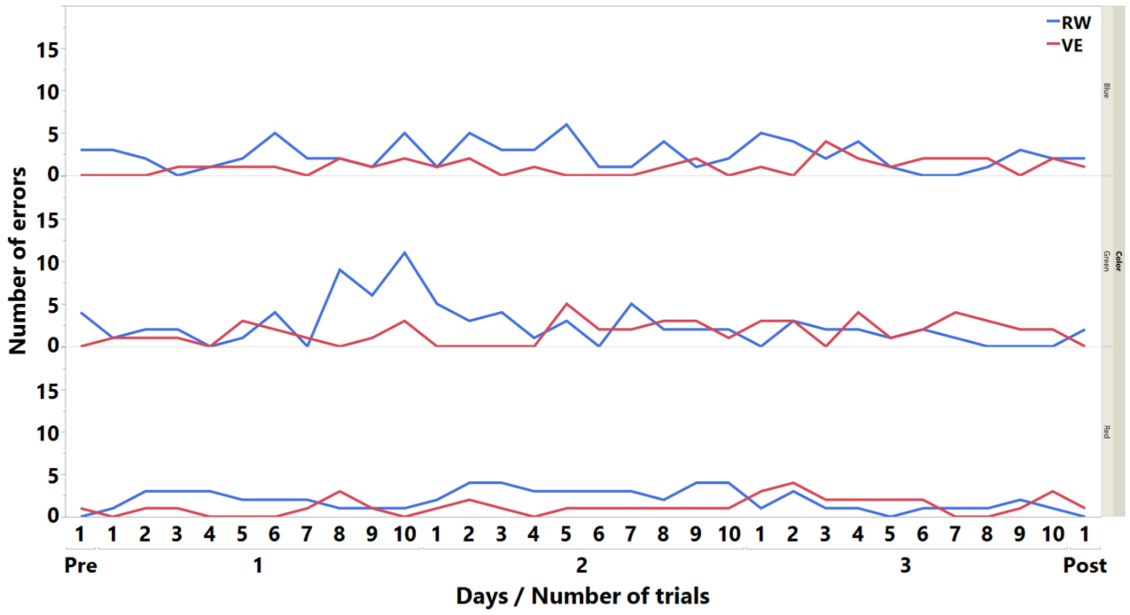

Figure S8. Participant 4 Number of Errors Results

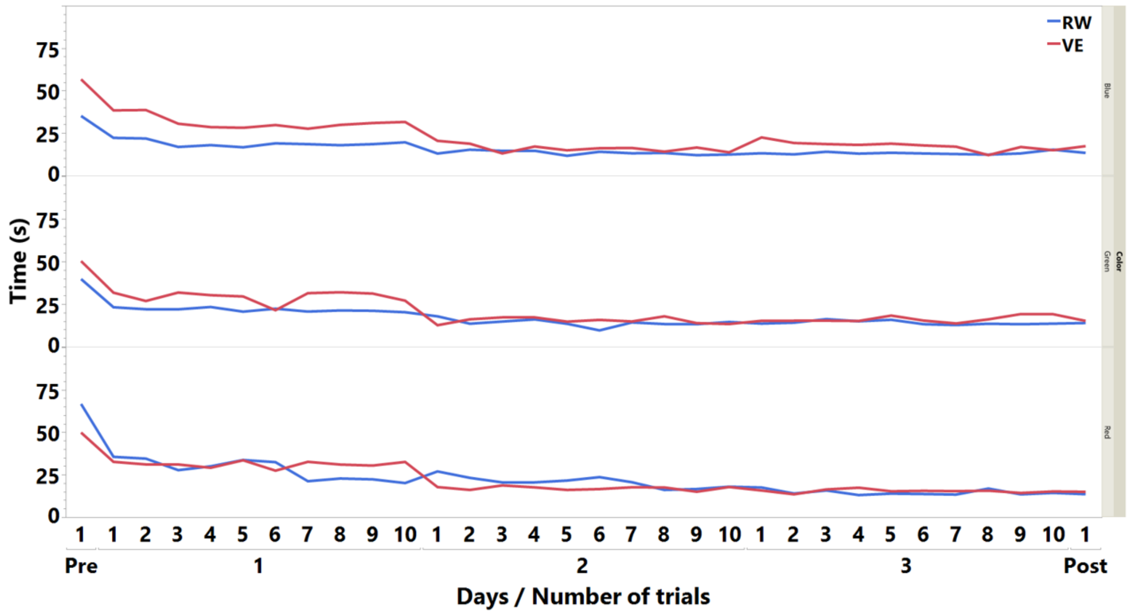

Figure S9. Participant 5 Time Results

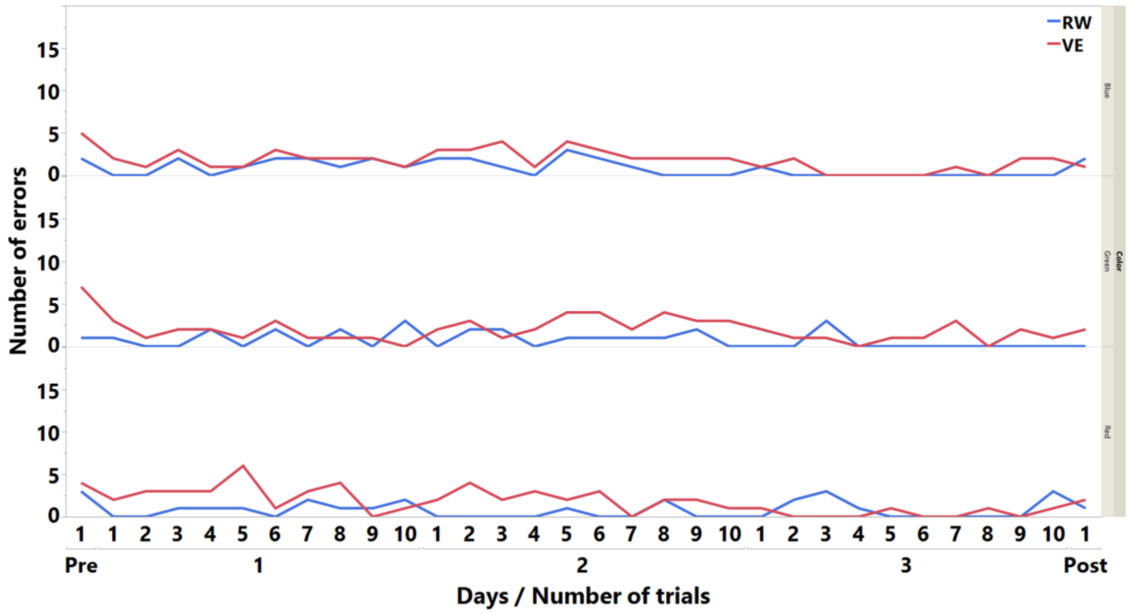

Figure S10. Participant 5 Number of Errors Results

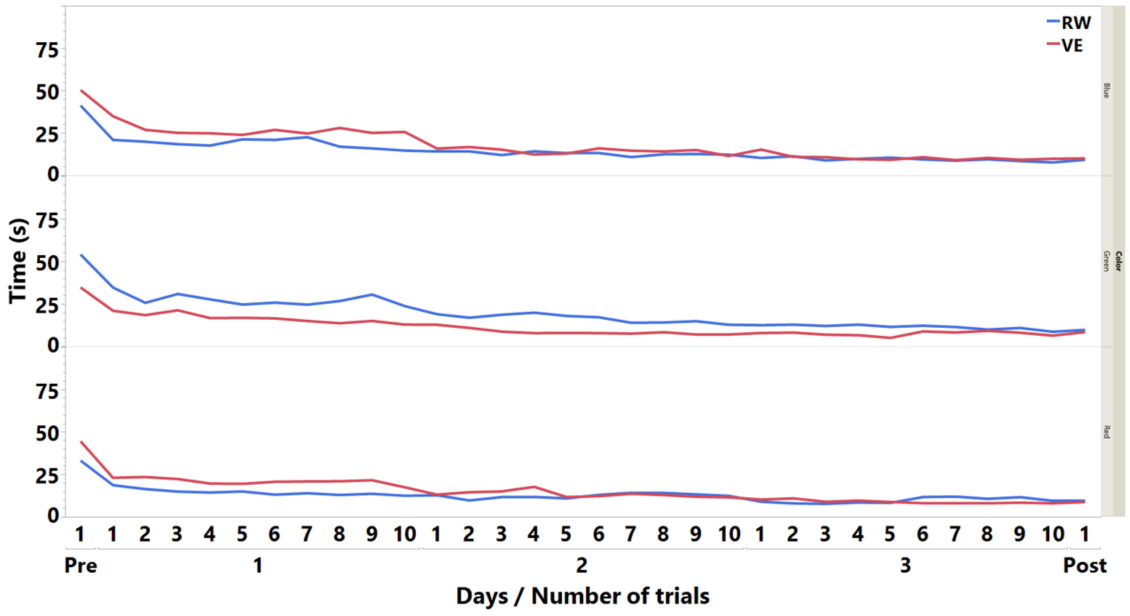

Figure S11. Participant 6 Time Results

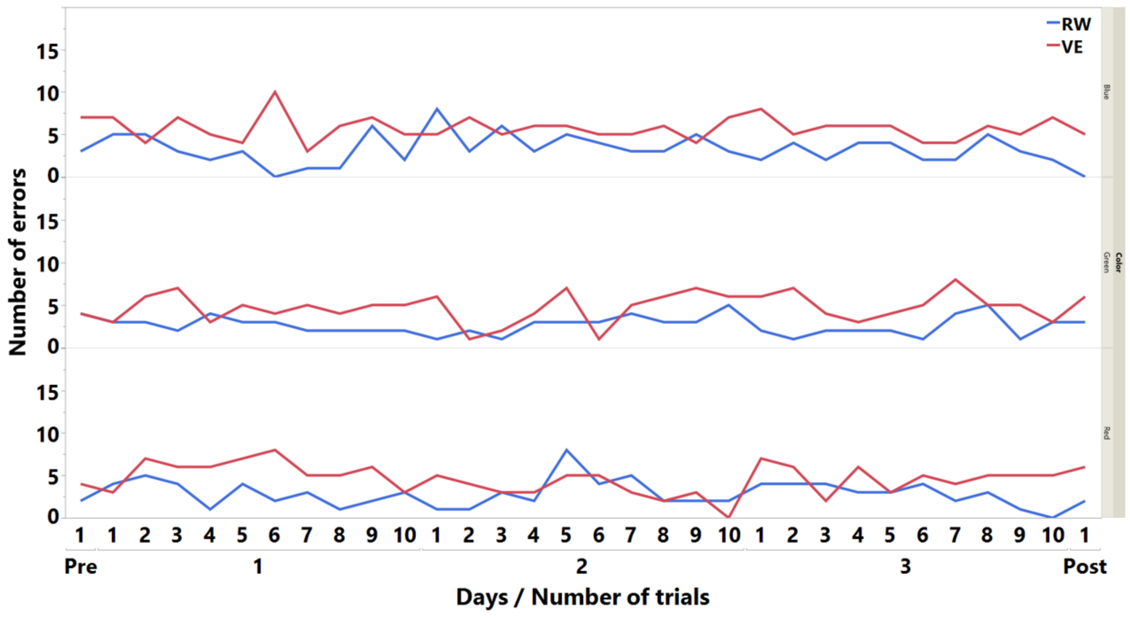

Figure S12. Participant 6 Number of Errors Results

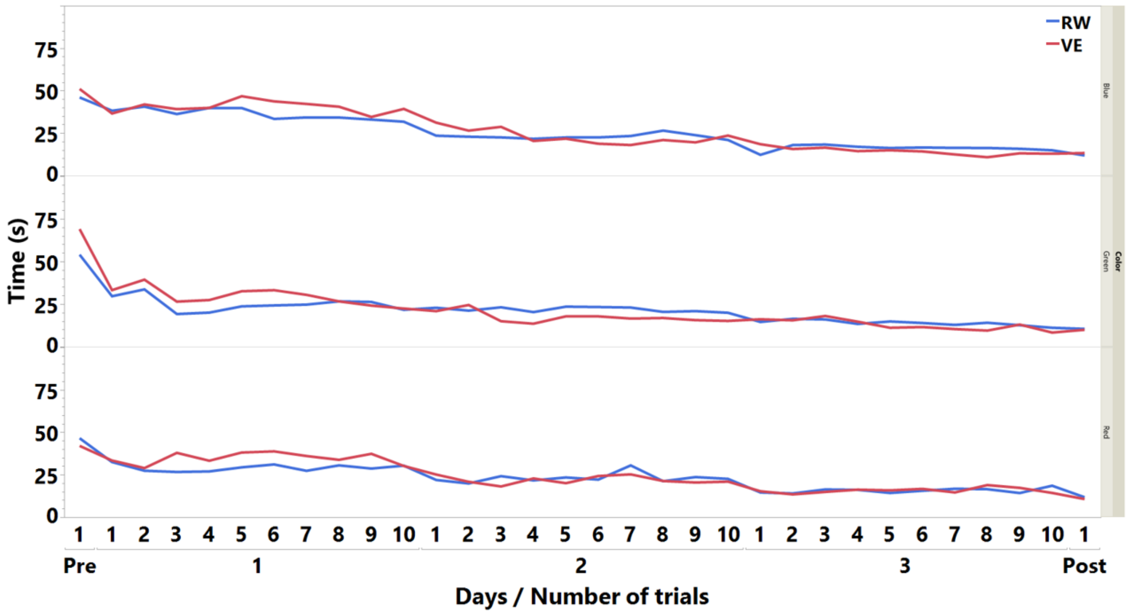

Figure S13. Participant 7 Time Results

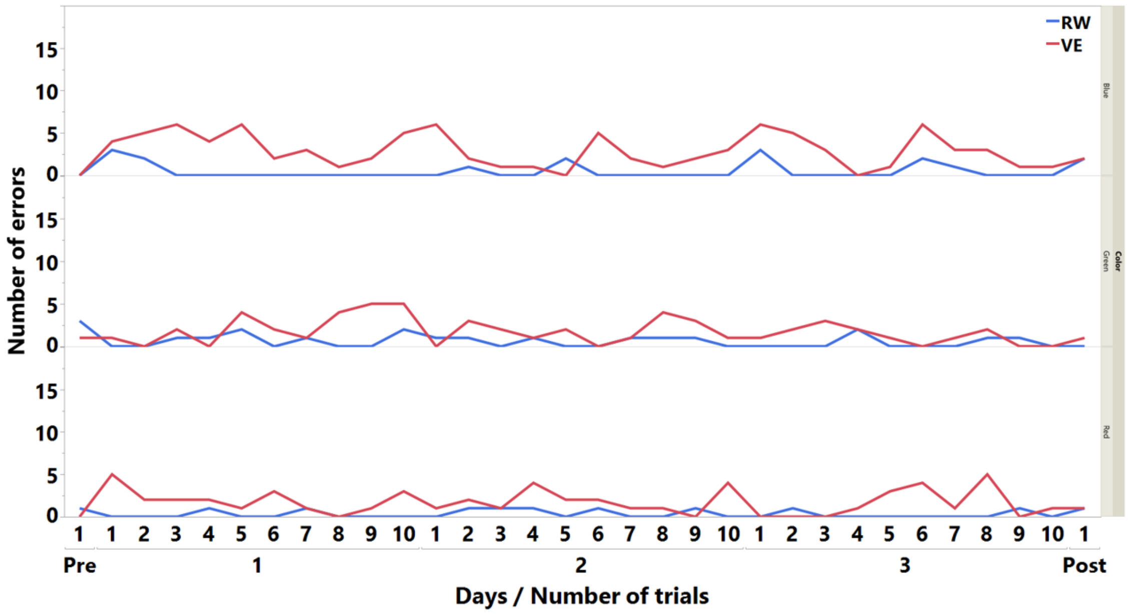

Figure S14. Participant 7 Number of Errors Results

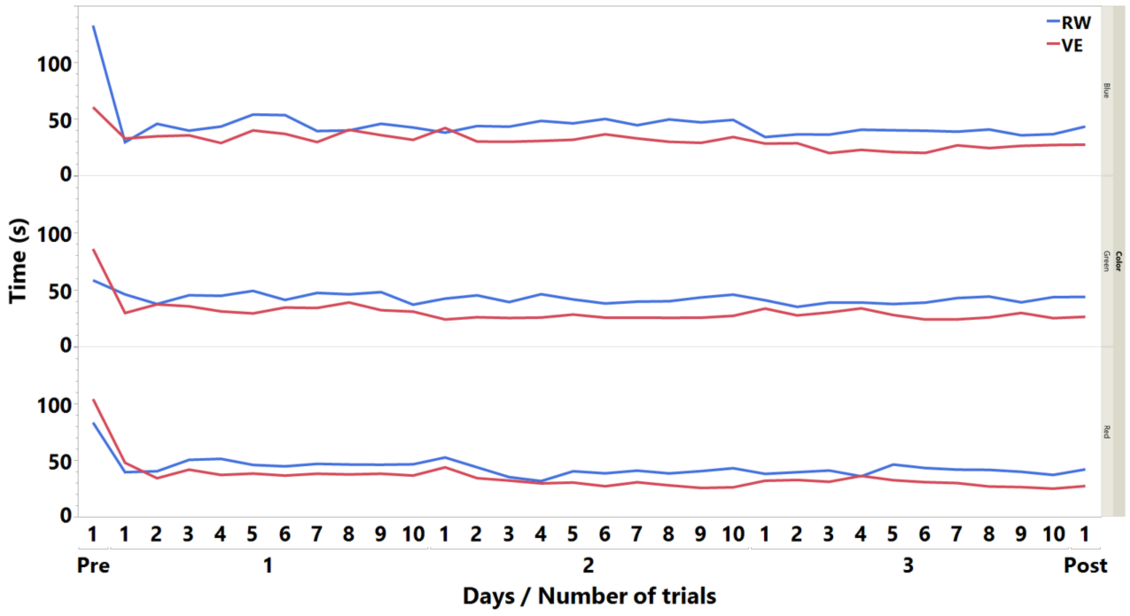

Figure S15. Participant 8 Time Results

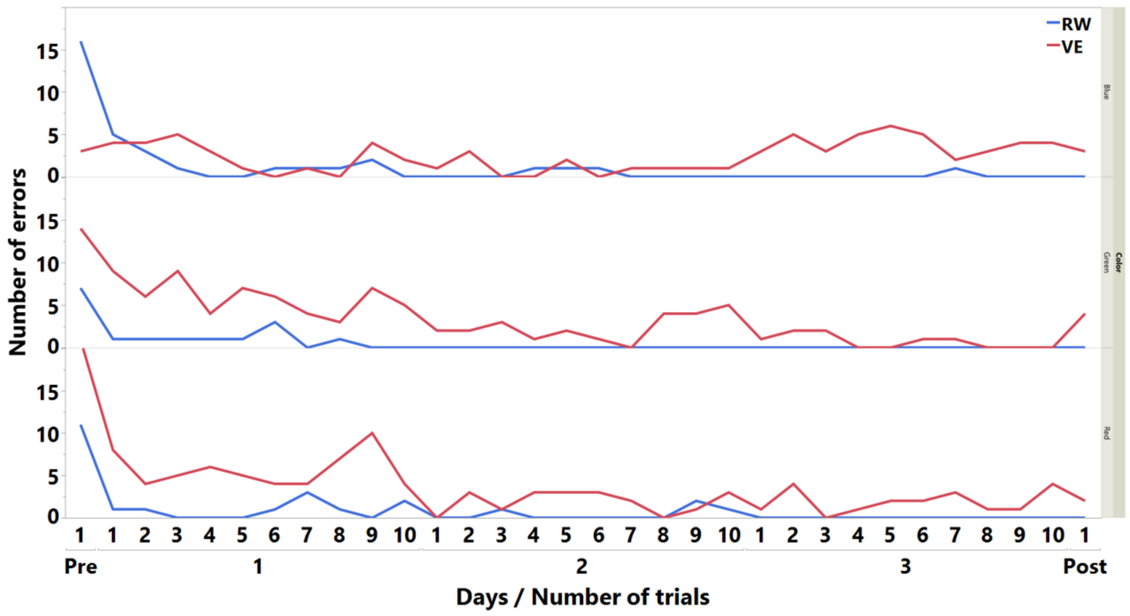

Figure S16. Participant 8 Number of Errors Results

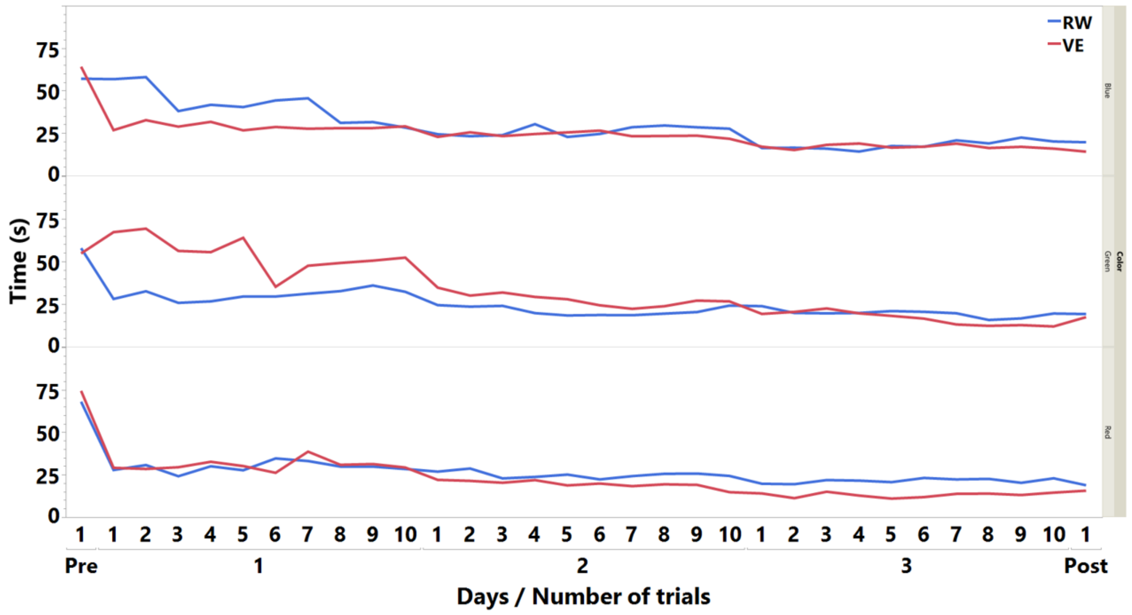

Figure S17. Participant 9 Time Results

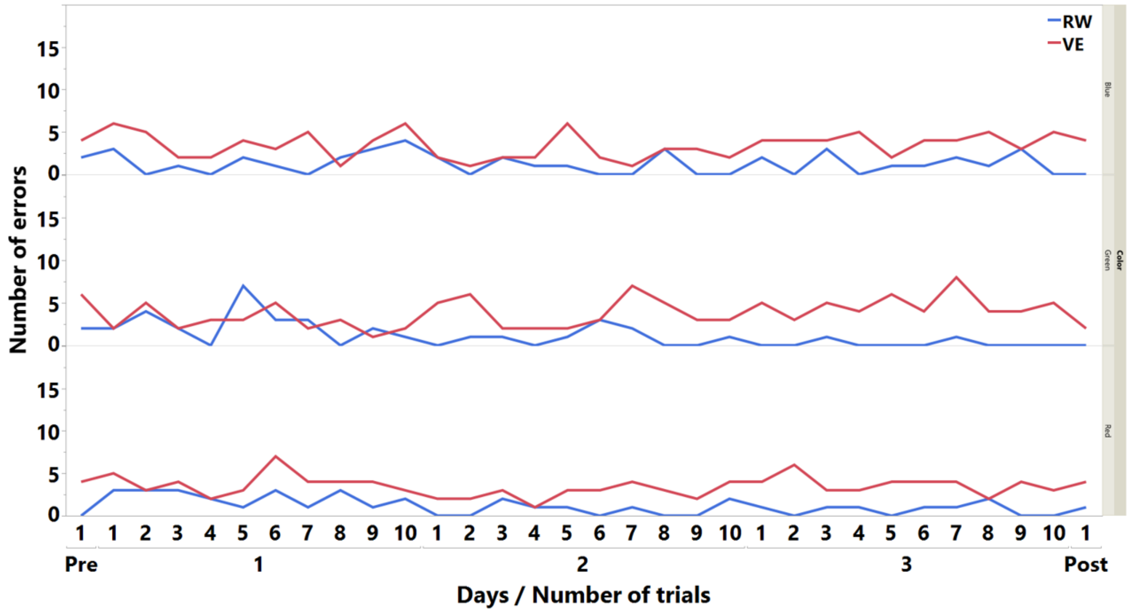

Figure S18. Participant 9 Number of Errors Results

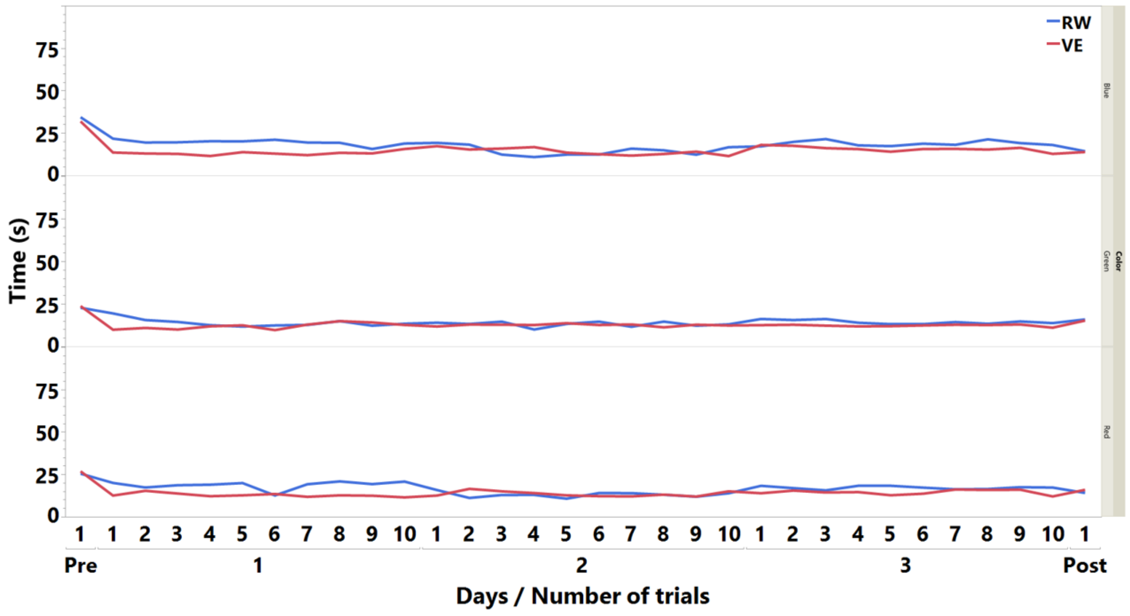

Figure S19. Participant 10 Time Results

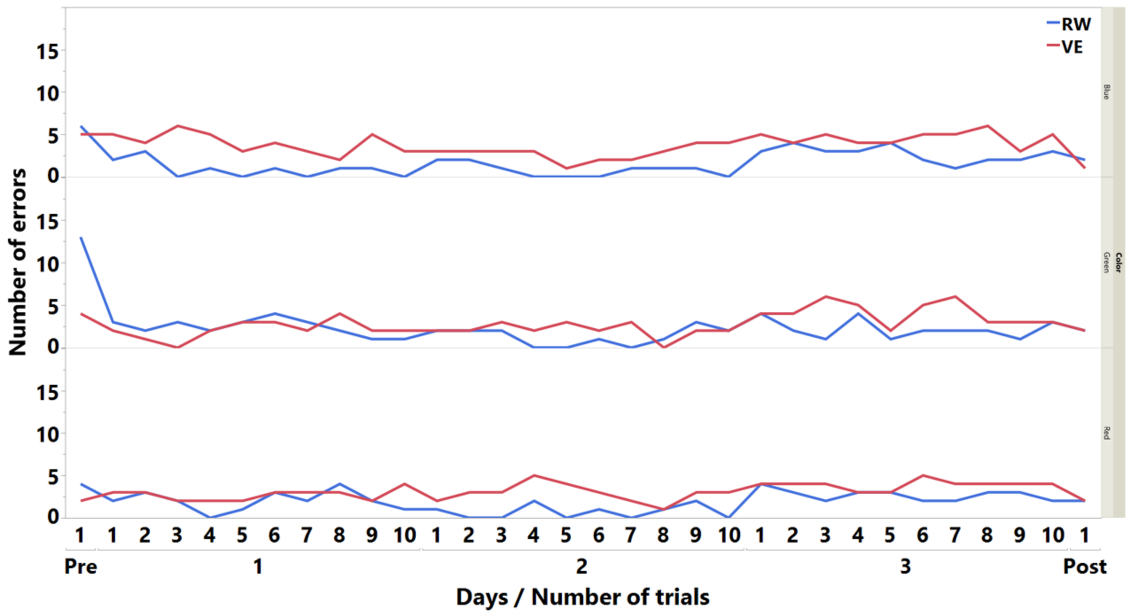

Figure S20. Participant 11 Number of Errors Results

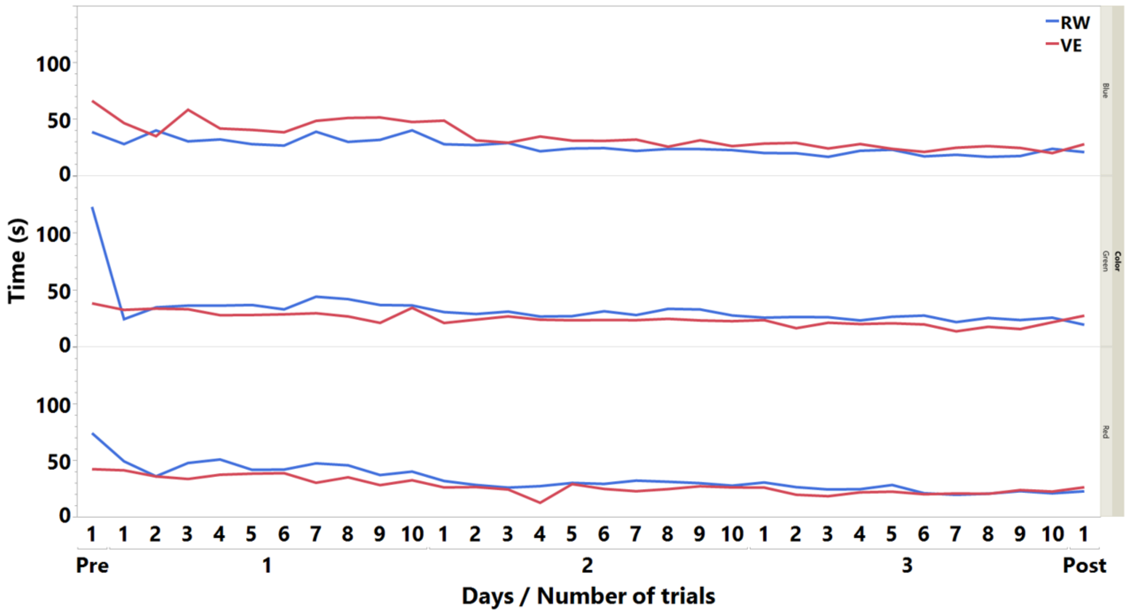

Figure S21. Participant 11 Time Results

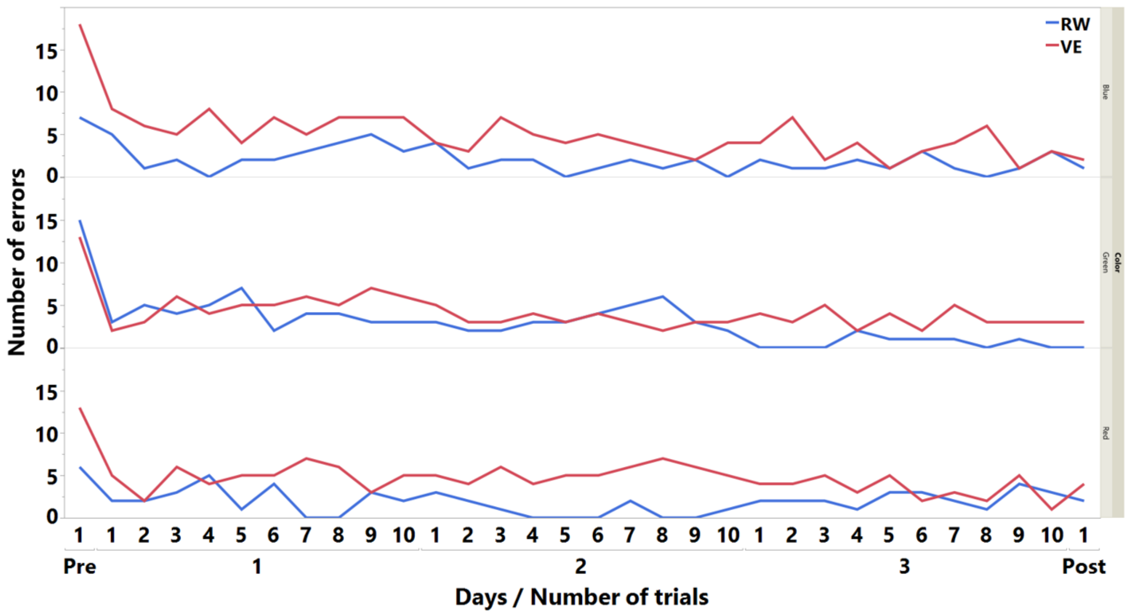

Figure S22. Participant 12 Number of Errors Results

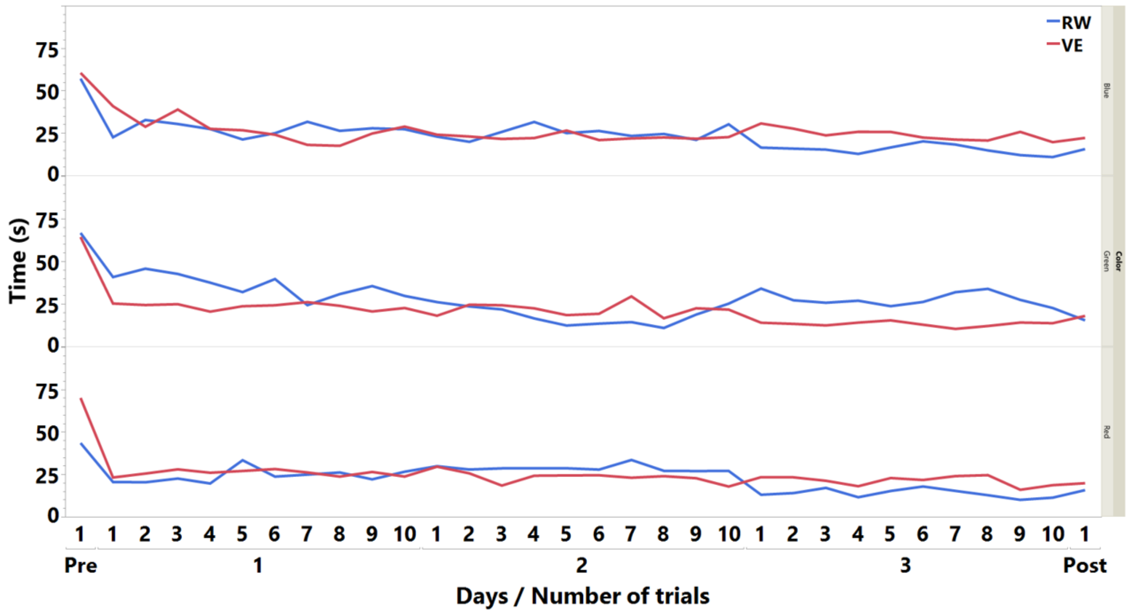

Figure S23. Participant 12 Time Results

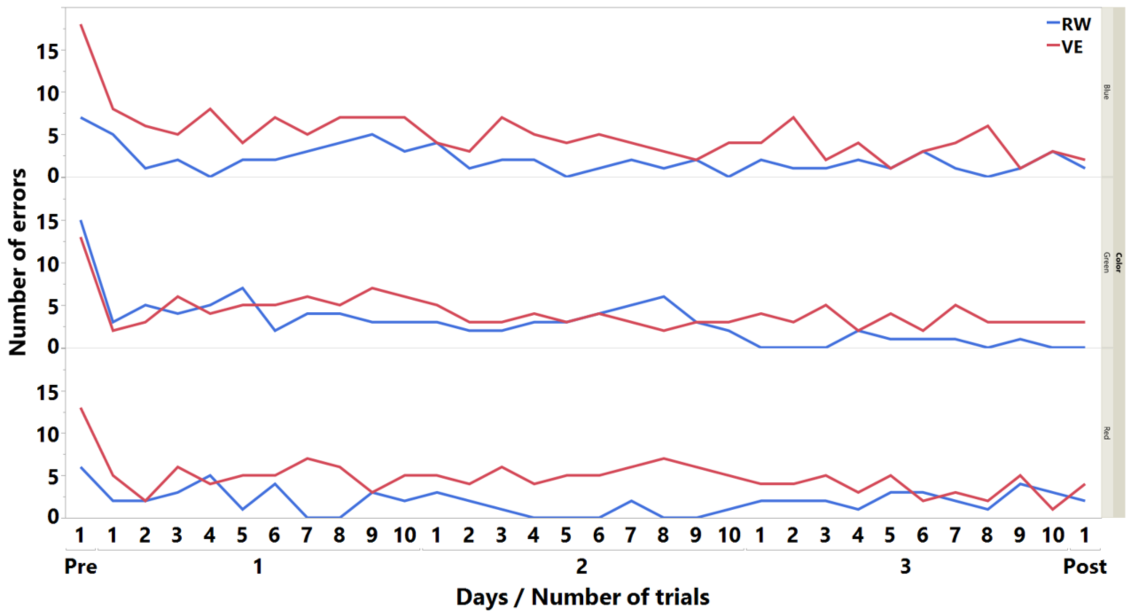

Figure S24. Participant 12 Number of Errors Results
